# Enhancement of vitality and activity of a plant growth-promoting bacteria (PGPB) by atmospheric pressure non-thermal plasma

**DOI:** 10.1038/s41598-018-38026-z

**Published:** 2019-01-31

**Authors:** Sang-Hye Ji, Ju-Sung Kim, Choong-Hwan Lee, Han-Sol Seo, Se-Chul Chun, Jaesung Oh, Eun-Ha Choi, Gyungsoon Park

**Affiliations:** 1 Plasma Bioscience Research Center, Kwangwoon University, Seoul, 01897 Republic of Korea; 2 Department of Bioscience and Biotechnology, Konkuk University, Seoul, 05029 Republic of Korea; 3 Department of Bioresources and food science, College of Life and Environmental Sciences, Konkuk University, Seoul, 05029 Republic of Korea; 4 Plasma Technology Research Center, National Fusion Research Institute, Gunsan-si, Jeollabuk-Do, 54004 Republic of Korea; 5 Department of Electrical and Biological Physics, Kwangwoon University, Seoul, 01897 Republic of Korea

## Abstract

The inconsistent vitality and efficiency of plant growth promoting bacteria (PGPB) are technical limitations in the application of PGPB as biofertilizer. To improve these disadvantages, we examined the potential of micro Dielectric Barrier Discharge (DBD) plasma to enhance the vitality and functional activity of a PGPB, *Bacillus subtilis* CB-R05. Bacterial multiplication and motility were increased after plasma treatment, and the level of a protein involved in cell division was elevated in plasma treated bacteria. Rice seeds inoculated with plasma treated bacteria showed no significant change in germination, but growth and grain yield of rice plants were significantly enhanced. Rice seedlings infected with plasma treated bacteria showed elevated tolerance to fungal infection. SEM analysis demonstrated that plasma treated bacteria colonized more densely in the broader area of rice plant roots than untreated bacteria. The level of IAA (Indole-3-Acetic Acid) and SA (Salicylic Acid) hormone was higher in rice plants infected with plasma treated than with untreated bacteria. Our results suggest that plasma can accelerate bacterial growth and motility, possibly by increasing the related gene expression, and the increased bacterial vitality improves colonization within plant roots and elevates the level of phytohormones, leading to the enhancement of plant growth, yield, and tolerance to disease.

## Introduction

Rapidly increasing population, global warming, and environmental pollution have become emerging threats to modern agriculture, resulting in food shortages worldwide. The world faces the need to develop sustainable and eco-friendly methods to improve agricultural productivity. The use of plant growth promoting bacteria (PGPB) as biofertilizer has been suggested as a replacement for existing methods, such as pesticides, herbicides and fungicides. PGPB are the bacteria living in or on plant roots and enhance plant productivity through various plant growth promoting activities, such as nitrogen fixation, efficient nutrients use by plant, hormonal modulation, and inhibition of the growth of harmful microbes^1^. Several groups of PGPB are known based on their nature and function, such as nitrogen fixer, phosphate solubilizer and mobilizer, micronutrient fertilizer, and biocontrol agent^1–4^. Nitrogen-fixing bacteria belonging to PGPB can fix atmospheric nitrogen and supply it to plants. Bio inoculants composed of nitrogen fixing bacteria are currently being used as an alternative to nitrogen fertilizers^5–7^. *Bacillus*, *Enterobacter*, and *Corynebacterium* have been reported as nitrogen fixing PGPB that improve plant growth and health through symbiosis with plants^8,9^.

Practical use and commercialization of PGPB as biofertilizers in agriculture started worldwide in the 1950s^10^. The current biofertilizer market occupies about 5% of the total chemical fertilizer market, and nitrogen fixing microbes are major components in biofertilizers^11^. Biofertilizers using PGPB have demonstrated several advantages in agricultural practices, such as environmentally friendly nutrition, improvement of soil fertility, and regulation of biotic and abiotic stresses^10,12,13^. However, the active use of PGPB sometimes encounters challenges in agricultural application, because of the inconsistent efficiency and vitality of PGPB^14^. First, PGPB is not broadly efficient to many plant species, and therefore field application can generate inconsistent outcomes in productivity. In particular, the surrounding environment and microbial community can affect the activity of PGPB. Secondly, colonization of PGPB on and in plants is not always stably maintained, because changeable environments and plant species can affect the success of PGPB establishment^15^. Finally, massive production can be challengeable for many isolated PGPB species, because of their poor growth in the culture system, compared to the natural habitat. Genetic engineering tools have been applied to improve the limitation of PGPB utilization. Genes encoding proteins that improve the beneficial function of PGPB are often introduced into the PGPB strains, and enhancement of bacterial efficiency is observed^16–18^. However, genetically engineered strains may be able to exert unfavorable influence on the soil microbial community, and in some strains, their successful establishment in plant is still an issue.

Atmospheric pressure non-thermal plasma has been suggested as a potential tool to improve the vitality and functionality of PGPB. Plasma is known as the 4^th^ state of material, in other words, ionized gas^19^. Plasma is one of the new technologies that are environmentally friendly and sustainable, and in recent years, has been applied in a variety of agricultural fields^20^. Studies have shown that plasma positively affects seed germination, plant development, and plant resistance^21–34^. In addition, non-thermal plasma-activated water (PAW) can deactivate various microorganisms^35–38^ and be used as fertilizer^27^. Plasma has demonstrated a broad spectrum of effects (from inactivation to activation) on the biological target samples, depending on the dose and the reactive species generated from the plasma. However, the potential of plasma in the activation of beneficial microorganisms has rarely been examined.

Enhancement of the vitality and functional activity of isolated PGPBs may be essential for agricultural sustainability. Currently, the Nagoya protocol on access and benefit sharing of biological resources is effective worldwide, and economic burden increases in using biological resources from other countries. Hence, improvement of the activity of available microbes becomes more important than ever. In this study, we investigated the potential of plasma to enhance the vitality and functional activity of a PGPB, in order to eventually promote plant growth, development, and stress regulation. *Bacillus subtilis* CB-R05 (PGPB) that was previously isolated from Korean rice cultivar as a nitrogen fixing endophytic bacterial strain^39^ was used in this study. Functional improvement of this bacterial strain by plasma was examined.

## Results

### Physical characterization of burst pulse type micro-DBD plasma

**Figure 1:**
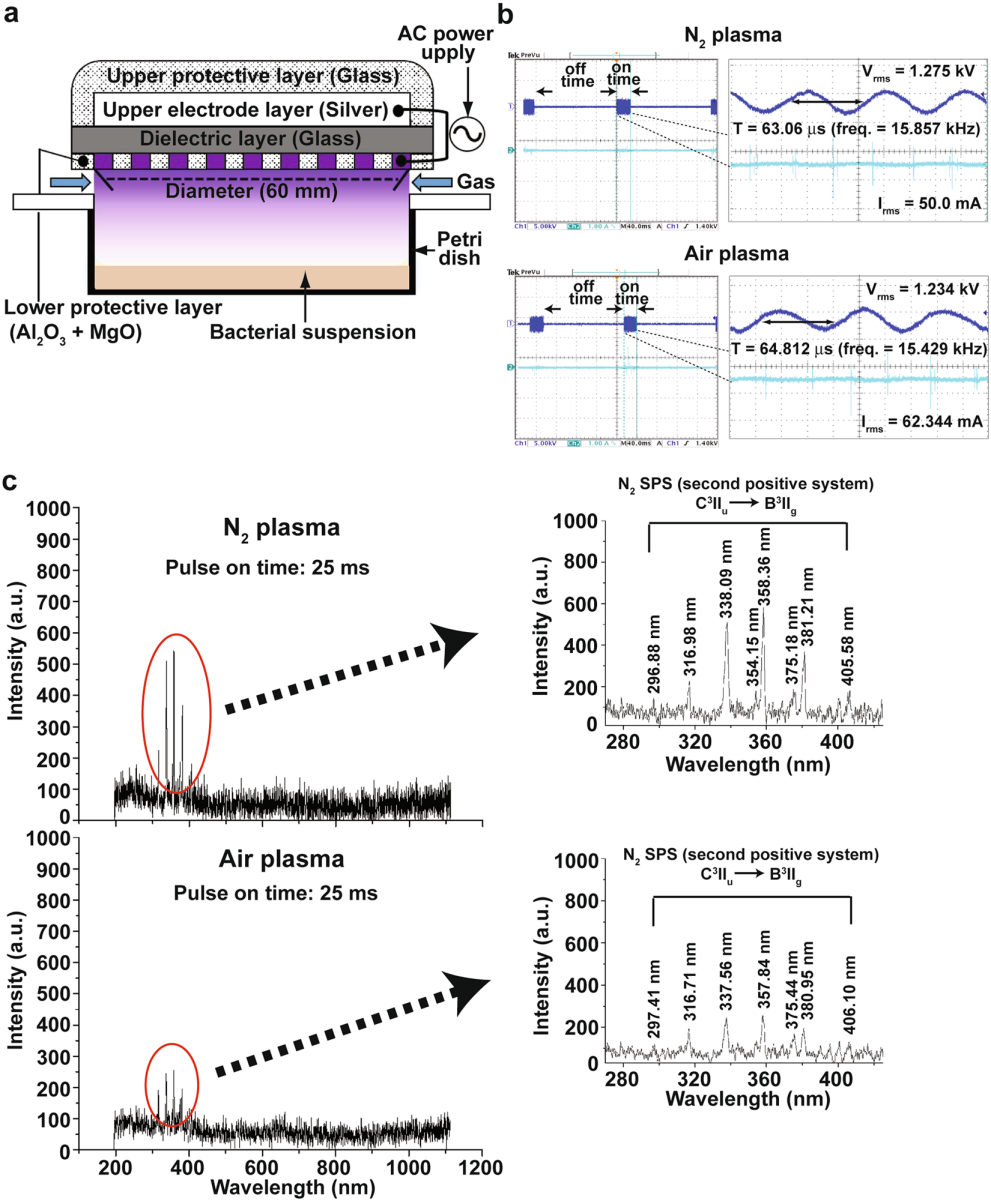
Structure and physical properties of micro DBD plasma. (**a**) Schematic view of micro DBD plasma device. (**b**) Current and voltage profile during plasma discharge. (**c**) Optical Emission Spectra (OES) of N_2_ and air plasma.

We used a micro-DBD plasma device modified from the previous version^40^ in the study. In this new version of the plasma device, plasma was generated using burst pulses of high voltage (Fig. 1a), and plasma energy was controlled by changing the duty ratio of burst pulses (on-and-off time ratio). The use of burst pulses could prevent the dielectric barrier covering electrodes from being damaged by heat produced during discharge, due to the very narrow gap between electrodes (of several hundred micrometers). By using burst pulses, less heat can be generated in the plasma source.

Figure 1b shows the applied voltage and current measured using digital oscilloscope during plasma discharge. A sinusoidal signal was detected in the burst pulse for both air and nitrogen plasma. The root mean square voltage and current were about 1.27 kV and 50 mA for nitrogen plasma, and about 1.23 kV and 62.3 mA for air plasma, respectively (Table 1). The frequency of waveform is about 15–16 kHz for both nitrogen and air plasma (Table 1). Plasma was periodically generated every 160 ms with 28.8 and 24.8 ms duration for nitrogen and air plasma, respectively (Table 1). Therefore, the duty ratio (%), defined as the ratio of ON time in one period of burst pulse, was 15 and 13% during nitrogen and air plasma discharge, respectively (Table 1). The electrical power (P) was calculated using the voltage and current waveforms, and specific duty ratio:

1 $${\rm{P}}={\rm{D}}\times \frac{1}{T}{\int }_{0}^{T}V(t)\cdot I(t)\,dt\ldots $$

where, D is the duty ratio for the duration time of the burst pulse, T is the period of the voltage signal, *V*(*t*) is the voltage signal and *I*(*t*) is the current signal for time behavior. Voltage V(t) and current I(t) are not the root mean square values, but the corresponding values according to each time from 0 to period T. The calculated electrical discharge energy was about (1.54 and 1.45) W for nitrogen and air plasma, respectively.

**Table 1:** Physical characterization of micro DBD plasma with burst pulse type.

	N_2_	Air
Voltage (V_rms_; kV)	1.276	1.234
Current (I_rms_; mA)	50	62.344
On time (ms)	28.8	24.8
Off time (ms)	160	160
Period (T; µs)	63.06	64.812
Frequency (freq; kHz)	15.857	15.429
Duty ratio of on time pulse	15%	13%
Energy transferred per second (W)	1.55	1.46

The optical emission spectra of plasma exhibited peaks corresponding to the excited nitrogen species of transitions referred to the second positive system (C^3^Π_u_ → B^3^Π_g_) (Fig. 1c). Both nitrogen and air plasma have the same excited nitrogen spectra with different intensity (Fig. 1c). No other transition lines were observed.

### Plasma enhances multiplication and motility of *B*. *subtilis* CB-R05

**Figure 2:**
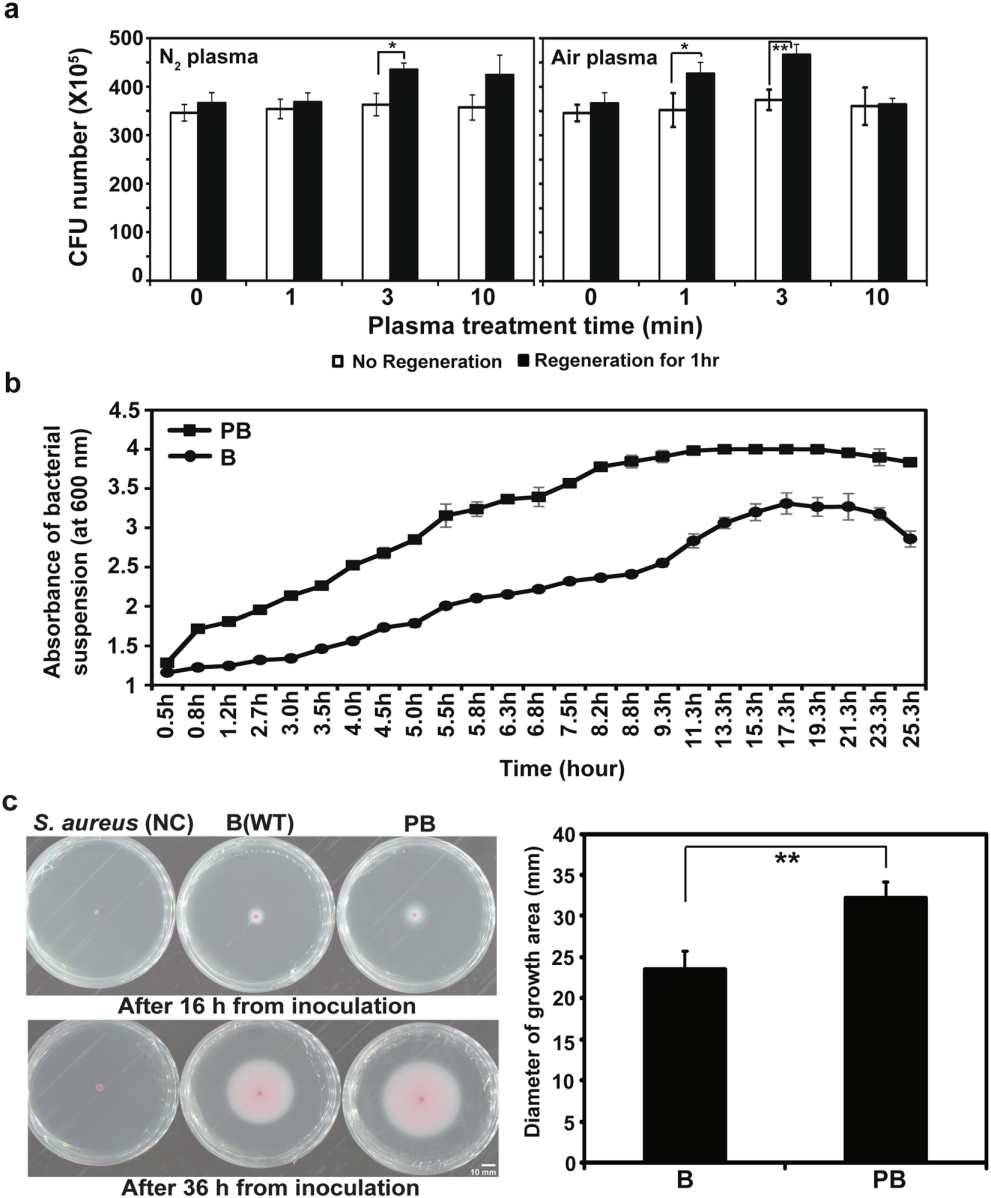
Bacterial vitality after plasma treatment. (Color in online). (**a**) CFU number of bacteria after N_2_ and air plasma treatment with or without regeneration in new media. (**b**) Comparison of bacterial growth measured as cell density (absorbance at 600 nm) during incubation time. (**c**) Bacteria incubated on Swarm assay media (LB medium, 0.5% agar with 1% TTC solution) plates for indicated time at 30 °C (left) and comparison of the diameter of swimming (moving) zone (right). *Staphylococcus aureus*, a non-motile bacterium, was used as a negative control. Pink color (left picture) is due to the production of formazan as a result of the reduction of TTC by bacterial metabolism, and therefore indicates the presence of live bacteria. B: untreated *B*. *subtilis* CB-R05, PB: plasma treated (air plasma for 3 min) *B*. *subtilis* CB-R05. All measurements were made in 3 or more than 3 replicates. *p < 0.05; **p < 0.01.

The colony forming unit (CFU) number of *B*. *subtilis* CB-R05 was not significantly changed right after treatment with plasma without regeneration (Fig. 2a). After regeneration in new media for 1 h, the CFU number of bacteria treated with N_2_ plasma for 3 and 10 min and air plasma for 1 and 3 min was significantly higher than that of the untreated bacteria (Fig. 2a). This indicated that multiplication of the bacterial cell was accelerated by both plasma treatments. When we monitored the concentration of bacterial cells over incubation time, the concentration of bacterial cells exposed to plasma was more rapidly increased over time than that of non-treated bacteria, as shown in the case of the 3 min air plasma treatment (Fig. 2b).

In Fig. 2c, a pinkish halo zone on the plate of *B*. *subtilis* CB-R05 indicates the movement of *B*. *subtilis* CB-R05, because it was not observed in non-motile bacterium *S*. *aureus*. The diameter of the pinkish halo zone was slightly greater in the plate of plasma treated bacteria than that of untreated bacteria after 16 h from inoculation (Fig. 2c). After 36 h, the diameter of the halo zone was significantly greater in the plate of plasma-treated bacteria than that of untreated bacteria (Fig. 2c). The longer the incubation period, the greater the difference in halo zone size between the plasma treated and untreated bacteria. The flagella motility of the plasma treated bacteria was significantly increased in a low concentration of agar medium, compared with non-plasma treated bacteria.

### Molecular changes in plasma treated bacteria

**Figure 3:**
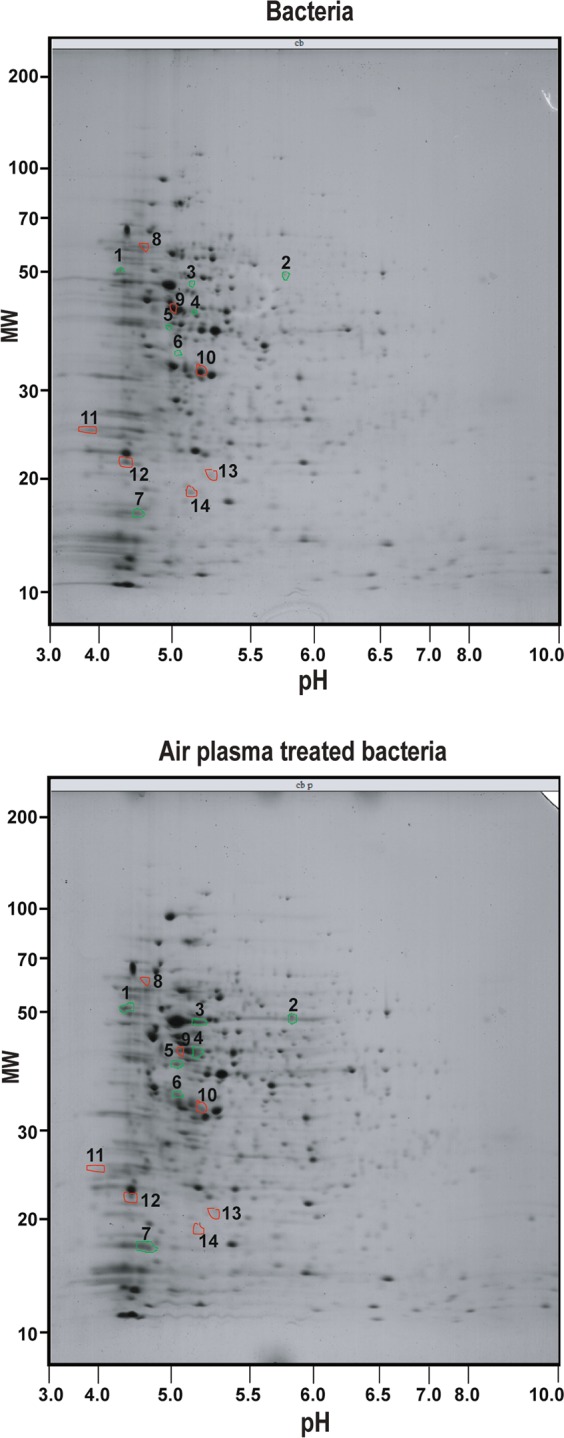
Two-dimensional electrophoresis of total proteins from bacteria. Total proteins were extracted from untreated and plasma treated (air plasma for 3 min) bacteria, and then applied to two-dimensional electrophoresis. The upper and bottom gels show 2-dimentional profiles of total proteins from untreated and plasma treated bacteria, respectively. Seven protein spots in which the level was increased in plasma treated bacteria more than 3-fold are indicated in green circles (numbered as 1–7) on both gels. Seven protein spots in which the level was decreased in plasma treated bacteria more than 3-fold are indicated in red circles (numbered as 8–14) on both gels. These 14 protein spots were used for protein identification.

To elucidate the molecular basis for the enhancement of growth and motility of CB-R05 by plasma, we first performed 2-dimensional electrophoresis to identify the difference in total protein profile between plasma treated and untreated bacteria. A total of 518 and 585 protein spots were observed on the gels of untreated and plasma treated bacteria, respectively (Fig. 3, Supplementary Table S1). Among these, 436 protein spots were paired between untreated (B) and plasma-treated (PB) bacteria (Supplementary Table S1). In the plasma treated CB-R05, 38 protein spots were up-regulated more than 2-fold, and 57 were down-regulated more than 2-fold, compared to the untreated bacteria (Supplementary Tables S1 and S2, Fig. S1). In addition, 82 and 149 protein spots were shown in the gel of only untreated bacteria and only plasma treated bacteria, respectively (Supplementary Tables S3 and S4, Fig. S2). We selected 14 protein spots for which the level was increased or decreased more than 3-fold and clarified the identity of those proteins using nano LC–MS/MS analysis (Fig. 3, Table 2). Except for 2 spots, 12 protein spots were identified (Table 2). The level of Ftsz protein (#4 spot in Fig. 3) known to be involved in cell division in *B*. *subtilis*^41^ was elevated more than 4-fold in plasma treated bacteria (Table 2). This observation may explain the enhancement of bacterial cell multiplication.

**Table 2:** Protein identification through MASCOT search.

Group No.	Ion score^a^	Mass (M_r_)	Cal. PI	Matched	Seq. coverage (%)	NCBI ID	Protein name
**Increased in plasma treated bacteria**							
1	488	50,531	5.05	17	22	gi|489324429	glutamine synthetase
2	404	52,730	5.54	23	18	gi|489316613	Aspartate ammonia-lyase
3	620	43,680	4.92	30	28	gi|489327768	elongation factor Tu
4	252	40,330	5.01	8	15	gi|158431169	Chain A, Ftsz
5	686	39,803	4.95	31	33	gi|1303956	YqjE
6	171	35,022	4.81	7	8	gi|489314681	Phosphate acetyltransferase
7	416	17,530	9.84	19	40	gi|143575	spc ORF1; S5
**Decreased in plasma treated bacteria**							
8							No significant results
9	1,222	46,503	5.03	89	45	gi|489322113	isocitrate dehydrogenase (NADP^+^)
10	1,039	32,448	5.17	59	60	gi|489324622	elongation factor Ts
11	98	22,882	9.88	4	8	gi|489244217	30S ribosomal protein S4
12	238	16,734	9.55	18	26	gi|351468459	50S ribosomal protein L6
13	83	17,241	5.15	5	10	gi|449028147	hypothetical protein C663_1583
14							No significant results

^a^Ions score is −10*Log(P), where P is the probability that the observed match is a random event. Individual ions scores >35 indicate identity or extensive homology (*p* < 0.05). Database: NCBInr (85,104,145 sequences; 31,181,569,036 residues). Taxonomy: *Bacillus subtilis* (133,129 sequences).

We also compared the profile of primary metabolites between untreated and plasma treated CB-R05. Our preliminary results of the analysis showed that the chromatograms of primary metabolites between untreated and plasma treated bacteria were not dramatically different (Supplementary Fig. S3). A total of 31 primary metabolites were identified, and the amounts of 8 metabolites among these were significantly increased or decreased (Supplementary Table S5, Fig. S4). The levels of oleamide, cyclohexanone, and phosphoric acid were significantly increased, whereas significant decrease in the levels of succinic acid, glycerol, oleanitrile, and stearic acid was observed in plasma treated bacteria (Supplementary Fig. S4b). Since phosphoric acid can be a good source for phosphorus, which is an essential nutrient in plant, the increased production of phosphoric acid in plasma treated CB-R05 may give a positive effect on rice growth.

### Plasma treated CB-R05 is more efficient in promoting the biomass and yield of rice plants

**Figure 4:**
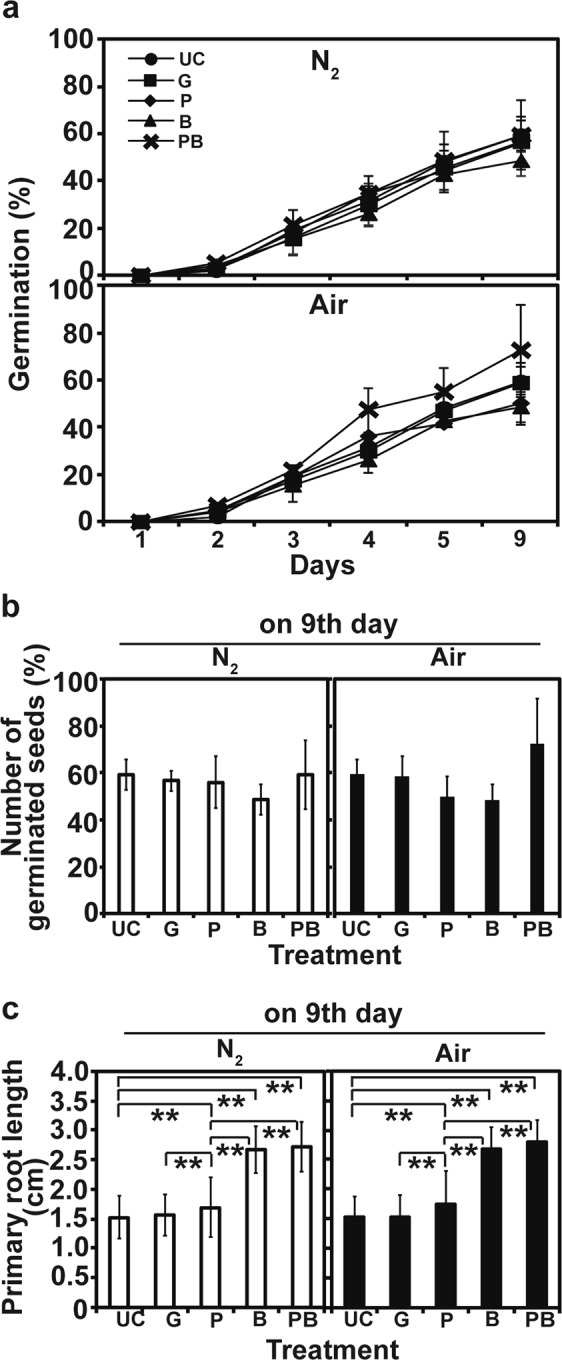
Germination of rice seeds after inoculation with plasma treated bacteria. (**a**) Percentage of daily seed germination. (**b**) The percentage of seed germination after 9 days. (**c**) Length of primary roots grown from germinated seeds. Each analysis for germination percentage included 50 seeds per treatment, and analysis was repeated three times. Total 90 seeds were used for measuring primary root length. All data points represent mean ± standard deviation of replicate measurements. **p < 0.01. UC: seeds inoculated with water, G: seeds treated with gas (N_2_ or air for 3 min), P: seeds treated with plasma (N_2_ or air plasma for 3 min), B: seeds inoculated with untreated *B*. *subtilis* CB-R05, PB: seeds inoculated with plasma treated (air plasma for 3 min) *B*. *subtilis* CB-R05.

Germination of rice seeds (*Ilpum*) was increased over time in all treatments, and no significant difference among treatments was observed (Fig. 4a). About 50–77% germination rate was observed in all treatments on Day 9, and the difference was not significant among treatments (Fig. 4b). The length of primary root was significantly greater in the germinated seeds inoculated with untreated (B) and plasma treated bacteria (PB), than water treated control seeds (UC) or seeds directly treated with plasma (P) (Fig. 4c). However, no significant difference was observed between seeds inoculated with untreated (B) and plasma treated bacteria (PB) (Fig. 4c).

**Figure 5:**
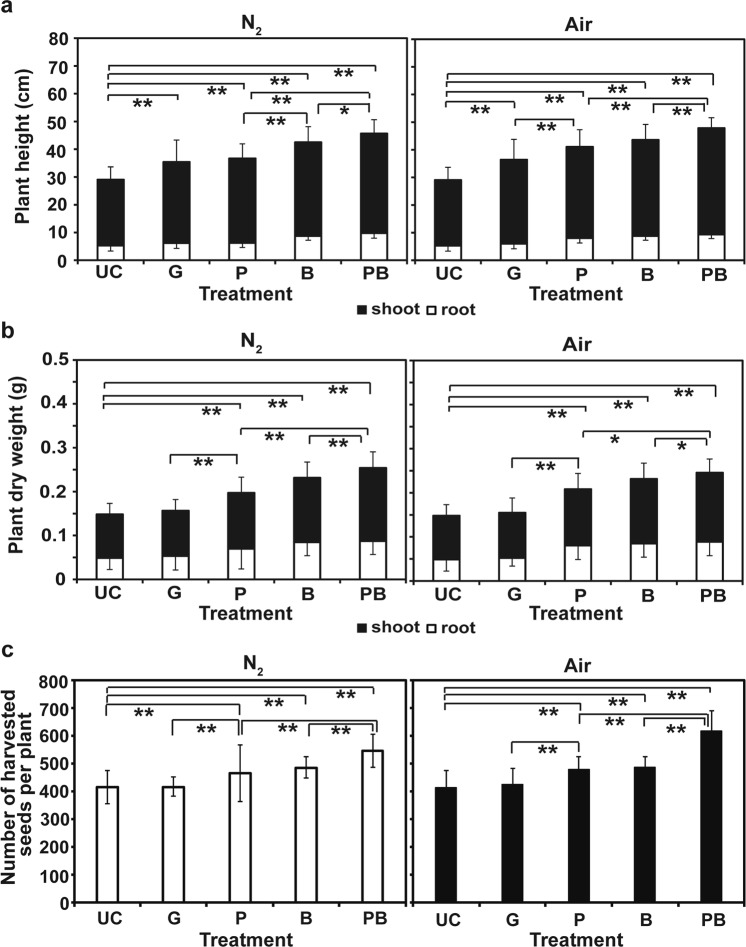
Growth and grain yield of rice plants. (**a**) Average length of shoot and root of individual rice seedling grown from germinated seeds for 6 weeks. (**b**) Average dry weight of individual rice seedling grown from germinated seeds for 6 weeks. (**c**) Average number of grains harvested from individual plant after 16 weeks. UC: seeds inoculated with water, G: seeds treated with gas (N_2_ or air for 3 min), P: seeds treated with plasma (N_2_ or air plasma for 3 min), B: seeds inoculated with untreated *B*. *subtilis* CB-R05, PB: seeds inoculated with plasma treated (air plasma for 3 min) *B*. *subtilis* CB-R05. A total of 150 plants per treatment were analyzed for height and dry weight. A total 95–107 plants per treatment were analyzed for grain yield. All data points represent the mean ± standard deviation of measurements. *p < 0.05; **p < 0.01.

The growth and yield of individual rice plant were analyzed after 6 and 16 weeks, respectively. Rice seedlings grown from seeds inoculated with plasma treated bacteria had the greatest height, dry weight, and grain yield among treatments (Fig. 5). Figure 5a shows that the height of rice seedlings infected with both N_2_ and air plasma treated bacteria (PB) was significantly greater than that of seedlings infected with untreated bacteria (B) and seedlings from direct plasma treatment (P). The average dry weight of seedlings infected with both N_2_ and air plasma treated bacteria (PB) was dramatically greater than that from other treatments (Fig. 5b). Significant increase in grain yield (number of harvested seeds per plant) was obtained with N_2_ (550 seeds/plant) and air (624 seeds/plant) plasma treated bacteria (Fig. 5c). Compared with the untreated bacteria, the average percentage of yield has increased more than 10%, when bacteria were treated with N_2_ and air plasma (Fig. 5c).

When seeds were directly treated with N_2_ and air plasma, the height, dry weight, and grain yield of the grown plants (P) were significantly higher than those of plants grown from gas treated seeds (G) or non-treated seeds (UC) (Fig. 5). However, no significant difference was mostly observed in the height, dry weight, and grain yield of plants between treatment with direct plasma (P) and bacteria (B) (Fig. 5). This indicates that inoculation with PGPB and direct plasma treatment may result in a similar level of effect on plant growth, and plasma treated PGPB improves the effectiveness beyond the effect level of PGPB.

### Plasma treated CB-R05 can increase the disease tolerance in rice plants

**Figure 6:**
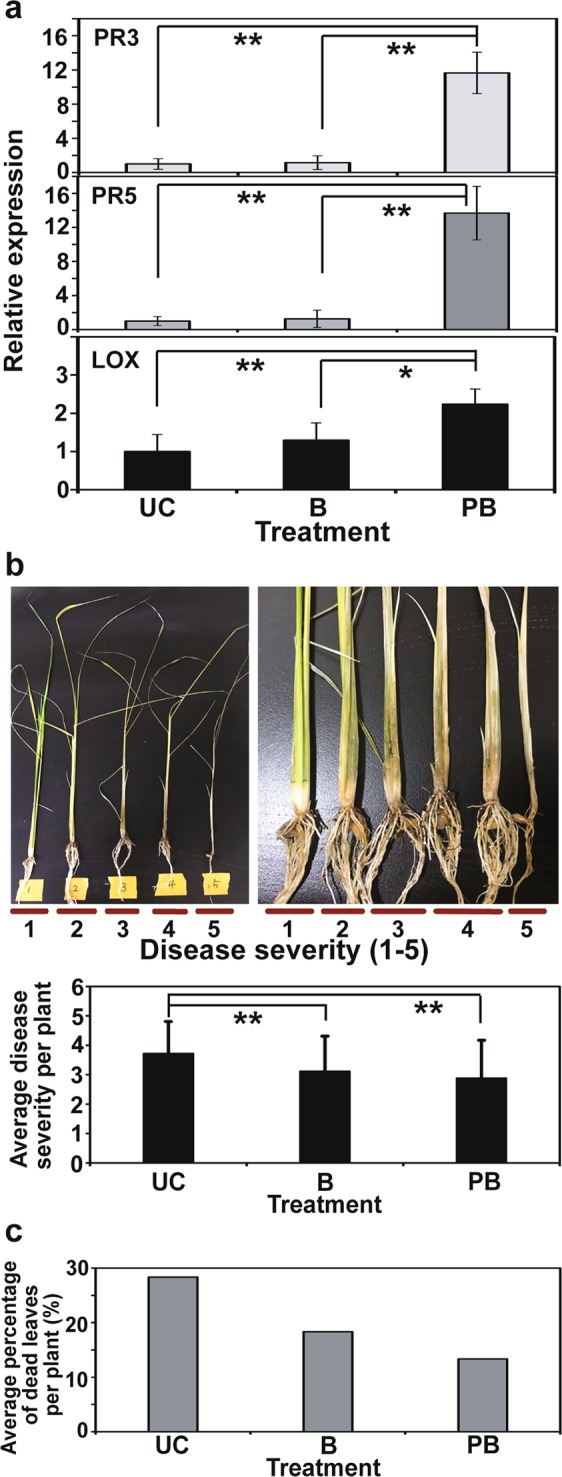
Tolerance of rice plants to fungal infection. (**a**) Expression of defense-related genes in rice seedlings (2-week-old) grown from the seeds inoculated with untreated and plasma treated *B*. *subtilis* CB-R05. The transcription levels of *OsPR3*, *OsPR5*, and *LOX* genes were examined by real-time RT-PCR. All data points represent the mean ± standard deviation of 5 replicate experiments. (**b**) Average level of disease severity in individual rice seedling inoculated with *R*. *solani* after 4 weeks. Disease severity was classified into 5 levels, and the standard for each level is described in the Materials and Methods. Each analysis included total 60 plants per treatment from 3 replicate batches (20 plants per replicate). (**c**) Average number of dead leaves from individual rice seedling inoculated with *R*. *solani* after 4 weeks. Each analysis included total 60 plants per treatment from 3 replicate batches (20 plants per replicate). UC: rice seedlings infected with water, B: rice seedlings infected with untreated *B*. *subtilis* CB-R05, PB: rice seedlings infected with plasma treated (air plasma for 3 min) *B*. *subtilis* CB-R05. *p < 0.05, **p < 0.01.

The transcription level of three PR genes was significantly increased in the 2-week-old rice seedlings infected with air plasma treated bacteria, compared to untreated bacteria or none (Fig. 6a). In particular, PR3 and PR5 genes were expressed more than 10-fold in seedlings infected with plasma treated bacteria than untreated bacteria and none (Fig. 6a).

After fungal pathogen *R*. *solani* AG-1 was inoculated onto the roots of 2-week-old seedlings, the disease development in plant was analyzed. After 4 weeks, the disease development was less severe in the rice plants infected with plasma treated bacteria than untreated bacteria or none (Fig. 6b, Supplementary Table S6). The untreated control plant (water inoculation) exhibited the highest disease severity among the three treatments (Fig. 6b, Supplementary Table S6). The number of dead leaves was reduced in the plants infected with plasma treated bacteria, compared to untreated bacteria and non-treated control (Fig. 6c, Supplementary Table S6). This indicates that inoculation with plasma treated bacteria can make the rice plant tolerant to *R*. *solani* pathogen.

### Plasma treatment enhances bacterial colonization and phytohormone production

**Figure 7:**
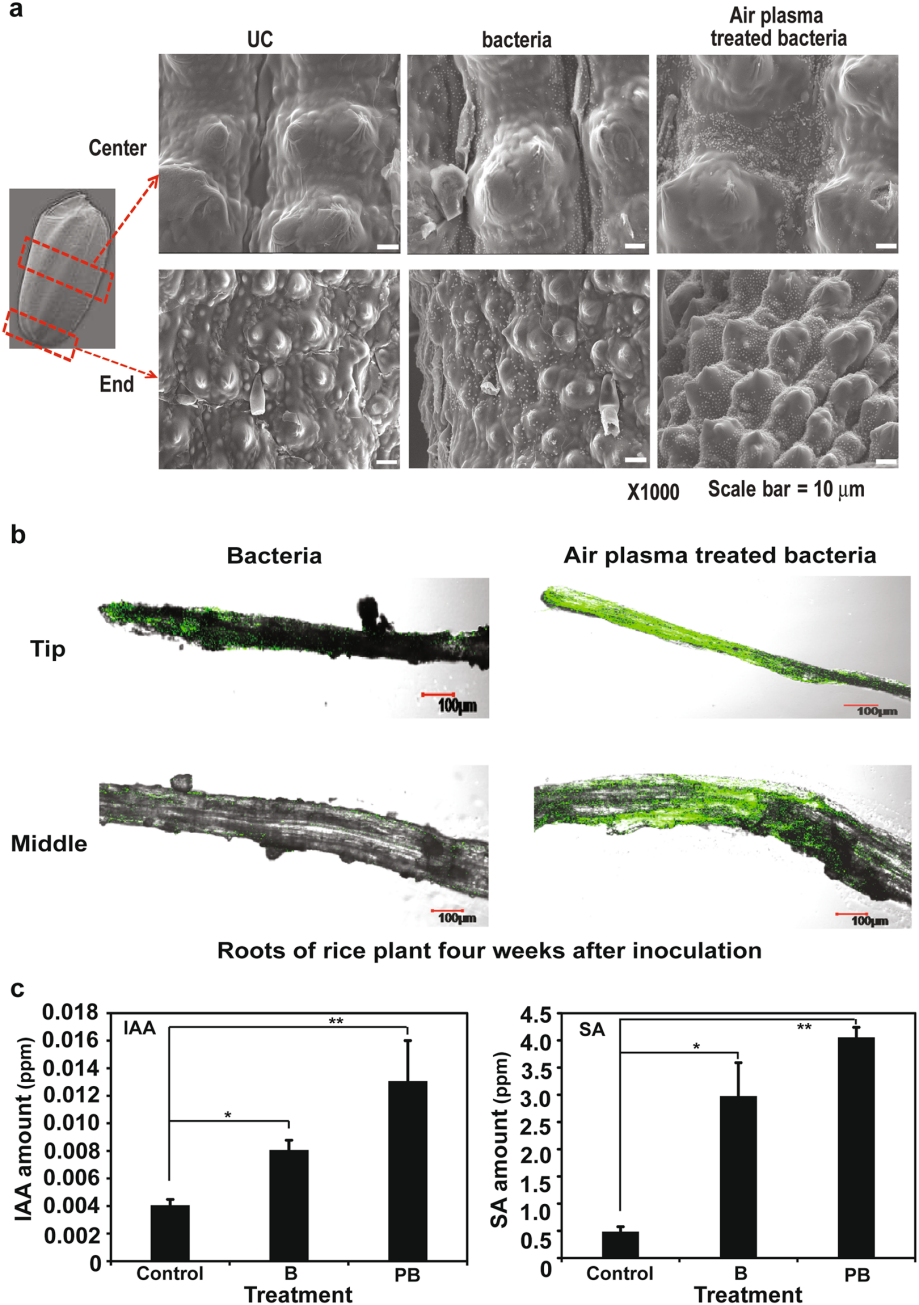
Attachment and colonization of bacteria and phytohormone production. (**a**) Comparison of bacterial attachment on rice seeds by SEM analysis. Bacteria were treated with micro DBD air plasma for 3 min. Sample observation was repeated three times and performed under 1,000× magnification. UC: seeds inoculated with water. (**b**) Confocal microscope images showing the colonization of *gfp*-tagged bacteria in rice root. Tip and middle part of root cross-section were observed. (**c**) Concentration of IAA and SA hormones was measured in rice seedlings grown from seeds inoculated with water (UC), untreated bacteria (B), and plasma treated (PB, air plasma for 3 min) bacteria.

To elucidate the mechanism for enhancing the functional efficiency of bacteria by plasma, we first examined the colonization of bacteria in plant. When seed surface was examined by SEM after inoculation with bacteria, slightly more bacteria were observed on the surface of seeds inoculated with plasma treated bacteria than untreated bacteria (Fig. 7a). Particularly, plasma treated bacteria were more densely crowded in the trough area of seed (Fig. 7a). Bacterial colonization within rice seedling roots was also examined by confocal microscope, using GFP labeled bacteria. Figure 7b shows that GFP fluorescence was more densely observed in a broader area of root infected with plasma treated bacteria than with untreated bacteria. This indicates that plasma treated bacteria can colonize better in rice plant root than untreated bacteria. Particularly, a large number of bacteria were distributed near the growth point and the vascular tissue in root (Fig. 7b). Plasma treated bacteria seem to colonize well within the rice roots, and to exist deep inside the roots.

The content of auxin (IAA), a phytohormone related to plant cell division and elongation, was increased in rice plants grown from seeds inoculated with bacteria, compared to plant grown from seeds inoculated with water (Fig. 7c). The IAA level was higher in plants infected with plasma treated bacteria than with untreated bacteria (Fig. 7c). The level of SA involved in the regulation of resistance to plant pathogens was higher in plants infected with plasma treated bacteria than with untreated bacteria or none (Fig. 7c). These results suggest that plasma treated bacteria can induce plant growth and disease resistance within rice plant more efficiently, compared to untreated bacteria.

## Discussion

Since the Nagoya protocol on access and benefit sharing of biological resources is effective worldwide, the improvement of efficiency in utilization of a limited number of microbial resources has become more important than ever. The efficiency of plant growth promoting bacteria (PGPB) or rhizobacteria (PGPR) as biofertilizer is dependent on their survival ability, competence, and interaction with other microbes in the environment (particularly soil), as well as their ability to promote plant growth. The atmospheric pressure non-thermal plasma technology that is demonstrated in our study can be a promising tool for enhancing the vitality and functional activity of PGPB through molecular and hormonal regulation. Studies have shown that plasma itself can promote seed germination and plant growth^22,26,28,29,32,42^. However, our study shows that PGPB seems to be equally or slightly more effective in the promotion of plant growth, compared to direct plasma treatment. This result indicates that the enhancement of activity of PGPB by plasma may be more efficient in increasing plant yield than PGPB itself and direct plasma treatment.

Plasma is known to have anti-microbial activity and used for sterilization in various situations^24,35–38,43^. However, our results suggest the activation effect of plasma on microorganisms. This may be possible due to the low dose of plasma treatment. Since ROS and RNS are generated by plasma, theoretically various outcomes ranging from inactivation to activation can be expected, depending on the level of ROS and RNS. The double-edged sword-like property (inactivation and activation) of plasma has been occasionally reported in studies^20,34,36,43,44^.

In respect of the mechanisms of enhancing plant growth, yield, and disease tolerance by plasma treated bacteria, our results point out that plasma treatment increases the multiplication and motility of bacterial cells, resulting in more successful colonization within roots (dense distribution in broader root area, as shown in Fig. 7a,b). It is highly possible that plasma generated reactive species have strengthened bacterial colonization. ROS is known to enhance bacterial colonization and biofilm formation by increasing the signal for quorum sensing which induces biofilm formation^45,46^. It is not clear whether plasma generated ROS is involved in regulating quorum sensing in our study. More intensive investigation will be needed for this. However, our results demonstrate that plasma may have enhanced the bacterial multiplication required for colonization. As shown in our 2-D gel electrophoresis result, the expression of a protein involved in cell division (Fstz) in *B*. *subtilis*^41^ is elevated by plasma treatment, and this can be a molecular evidence supporting the acceleration of multiplication. Increase in multiplication may contribute to the bacterial colonization. Plasma mediated change in the level of gene expression can lead to the activation of biological processes^47^. Since reactive species are well known signaling molecules^48,49^, ROS and RNS generated by plasma can possibly be key regulators in the expression of genes involved in bacterial multiplication and motility. However, our preliminary result also shows that plasma generated reactive species can cause base pair changes in the genomic DNA. Three base pair changes were observed in the genomic DNA of plasma treated bacteria (Supplementary Table S7). Since some of these changes do not lead to the change in amino acid sequence, or not all proteins are affected, the influence on the phenotype may be insignificant (Supplementary Table S7). In addition, this may result from the plasma dose, which can affect the degree of change (genetic or epigenetic), but more intensive investigations are needed on this matter.

Our result also shows that plasma treated bacteria enhance the level of phytohormones in plant. Elevation of growth regulators produced by plant and bacteria is often observed in PGPB infected plants^50,51^. In our study, the level of IAA and SA was increased in bacteria infected plants, and particularly, increased more in plants infected with plasma treated bacteria. The higher level of colonization of plasma treated bacteria within plant might have caused the increase in hormone level. It is not clear whether newly synthesized IAA is originated from bacteria or plant. However, the increase in IAA level must have contributed to the enhancement of plant growth. Interestingly, the level of SA hormone is also more increased in plants infected with plasma treated bacteria than untreated bacteria. Since SA is produced during the induced systemic resistance, the elevated SA level in plant infected with plasma treated bacteria is likely to cause the promoted PR gene expression and tolerance to fungal infection observed in our results.

Our preliminary analysis of primary metabolites in bacteria exhibits interesting points about the metabolic interaction between bacteria and plant. The phosphoric acid level increases in plasma treated bacteria. PGPBs are known to facilitate the solubilization of nutrients such as nitrogen, phosphorus, and iron in soil, and improve the nutrient use by plant^1,4,39^. Insoluble phosphorus present in soil can be soluble by the action of organic acids synthesized by PGPBs, and plants can absorb this soluble phosphorus. This is a known mode of action for the increase in availability of phosphorus in soil to plants by PGPBs. However, our result, the elevated production of phosphoric acid by plasma treated bacteria, can suggest another possibility that plasma treated bacteria can directly provide phosphorus to plants. Although secretion of phosphoric acid from bacteria is another issue requiring for intensive studies, plasma treated bacteria can possibly be useful for improving the nutrient acquisition by plants. In addition, the levels of oleamide and cyclohexanone were increased in plasma treated bacteria. Oleamide and cyclohexanone are known as anti-microbial agents^52–54^. If plasma treated bacteria can release these metabolites, they may be able to provide tolerance to plants against pathogen infection. Phosphoric acid, oleamide, and cyclohexanone produced highly by plasma treated bacteria can play roles in plant growth and disease defense, and therefore their location in plant may be related to their functions. The dense distribution of plasma treated bacteria around the growth point and vascular tissues within root indicates that these metabolites produced by plasma treated bacteria can be easily used for growth and transported to the other parts of plant, preparing the defense to pathogen attack.

In this study, we examined the potential of atmospheric pressure non-thermal plasma in enhancing the vitality and functional activity of a plant growth promoting bacteria (PGPB). Plasma treatment accelerated the bacterial growth and motility, possibly through the regulation of gene expression. These changes in bacteria may have promoted bacterial colonization in plant roots, and eventually elevated more production of phytohormones that stimulated plant growth and tolerance to fungal infection. Our results suggest that plasma can be a potential tool for improving the vitality and activity of beneficial microorganisms by regulating gene expression without dramatic genetic change.

## Methods

### Plasma device

A Micro-DBD (dielectric barrier discharge) plasma device with burst pulse type of high voltage inverter was used in this study. This device is a modified version of the micro-DBD plasma unit used in previous studies, in which the power system has been changed from continuous to burst pulse type^55^. The structure of microelectrodes in the plasma device is the same as that in the continuous pulse type DBD plasma unit (Fig. 1a), and well described in the previous study^55^. Plasma was generated using air or nitrogen gas with 1.5 Lpm (liter per minute) flow rate, and electric power of 1.2 kV input voltage and 50–63 mA current. The optical emission spectra of plasma were measured by spectrometry (Ocean Optics, HR4000) with coupled charged device (CCD).

### Treatment of bacteria with plasma and measurement of bacterial growth

A plant growth-promoting bacteria (PGPB), *Bacillus subtilis* CB-R05, isolated within rice plant root, was treated with micro DBD plasma. This bacterial strain is known in the previous study to effectively enhance growth of the rice cultivar *Ilpum*^39^. To treat bacteria with micro DBD plasma, bacterial cells were grown in tryptic soy broth (TSB) at 37 °C for 16 h, and then pelleted down by centrifugation at 3,134 × *g* for 5 min. Bacterial cell pellet was suspended in new TSB, and the concentration was adjusted to 1.0 optical density at 600 nm. Bacterial suspension (5 ml) was placed in a 35 mm petri dish, and then exposed to micro DBD plasma at a distance of 1 mm. The plasma-treated bacterial suspension was centrifuged at 3,134 × g for 5 min, and the bacterial pellet was washed with sterile distilled water. The bacterial cell pellet was resuspended in 5 ml of new TSB and regenerated for 0 (control) and 1 h. To measure the bacterial growth, the regenerated bacteria were spread onto tryptic soy agar (TSA) plates, and the CFU number was counted.

### Measurement of bacterial motility

Semi-solid agar media were used in the test for bacterial motility. The composition of the medium was as follows: in 1 L, 3 g beef extract, 10 g pancreatic digest of casein, 5 g sodium chloride, 5 ml of 1% triphenyltetrazolium chloride (TTC) solution, and 400 g agar (MB cell, Los Angeles, USA). After treatment with or without plasma, *B*. *subtilis* CB-R05 inoculum was spotted on the center of semi-solid agar media plate using an inoculum needle. Then, plates were incubated at 30 °C for 18 h. Bacterial motility was assessed by measuring the diameter of the area that bacteria were spread out from the center of the plate. *Staphylococcus aureus*, a non-motile bacterium, was used as negative control.

### 2D gel electrophoresis and protein identification

To analyze the global expression of proteins within bacterial cells, two-dimensional electrophoresis was carried out as previously described^56^. Total protein extraction, two-dimensional electrophoresis, and protein identification were performed at the Yonsei Proteomics Research Center (YPRC). The extracted proteins were suspended in sample buffer (7 M urea, 2 M thiourea, 4.5% CHAPS, 100 mM DTE, 40 mM Tris, pH 8.8). Aliquots in sample buffer were applied to immobilized pH 3–10 nonlinear gradient strips (Amersham Biosciences, Uppsala, Sweden). Isoelectric Focusing (IEF) was performed at 80,000 V per hour. The second dimension was analyzed on 9–16% linear gradient polyacrylamide gels (18 cm × 20 cm × 1.5 mm) at a constant 40 mA per gel for approximately 5 h. After protein fixation in 40% methanol and 5% phosphoric acid for 1 h, the gels were stained with CBB G-250 for 12 h. The gels were destained with H_2_O, scanned in a Bio-Rad GS710 densitometer (Richmond, CA, USA) and converted into electronic files, which were then analyzed using Image Master Platinum 5.0 image analysis program (Amersham Biosciences, Little Chalfont, UK).

To identify proteins, nano LC–MS/MS analysis was performed with a nano HPLC system (Agilent, Wilmington, DE, USA). The nano chip column (Agilent, Wilmington, DE, 150 mm × 0.075 mm) was used for peptide separation. The mobile phase A for LC separation was 0.1% formic acid in deionized water, while the mobile phase B was 0.1% formic acid in acetonitrile. The chromatography gradient was as follows: a linear increase from 3 to 45% B in 30 min, 45 to 95% B in 1 min, 95% B in 4 min, and 3% B in 10 min. The flow rate was maintained at 300 nL/min. Product ion spectra were collected in the information-dependent acquisition (IDA) mode and were analyzed by Agilent 6530 Accurate-Mass Q-TOF using continuous cycles of one full scan TOF MS from 300 to 2,000 *m*/*z* (1.0 s), plus three product ion scans from 150 to 2,000 *m*/*z* (1.5 s each). Precursor *m*/*z* values were selected starting with the most intense ion, using a selection quadrupole resolution of 3 Da. The rolling collision energy feature was used, which determines collision energy based on the precursor value and charge state. The dynamic exclusion time for precursor ion *m*/*z* values was 60 s. The mascot algorithm (Matrixscience, Boston, MA, USA) was used to identify peptide sequences present in a protein sequence database. Database search criteria were: *B*. *subtilis* fixed modification; carboxyamidomethylated at cysteine residues; variable modification; oxidized at methionine residues, maximum allowed missed cleavage; 2, MS tolerance; 100 ppm, MS/MS tolerance; 0.1 Da. Only peptides resulting from trypsin digests were considered.

### Inoculation of bacteria on rice seeds and assessment of plant vitality

For inoculation of rice seeds with plasma treated bacteria, rice seeds were first surface-sterilized by being immersed in 70% ethanol for 1 min, and then shaken in 1.2% (w/v) sodium hypochlorite (NaOCl) solution for 15 min. Rice seeds were washed three times with sterile deionized water by shaking (15 min each). For inoculation, 30 surface-sterilized rice seeds (cultivar *Ilpum*) were soaked in 10 ml suspension of plasma treated bacteria, non-treated bacteria, or deionized water at 30 °C for 1 h. Deionized water was used as inoculum for untreated control. We also treated 50 rice seeds directly with only gas and plasma (N_2_ and air), for comparison.

To assess the germination rate of rice seeds, 50 seeds from each treatment were placed on 2 layers of wet filter paper in 90 mm petri dishes, and the petri dishes were incubated in a plant growth chamber (25 °C, 50% moisture, 16 h light and 8 h dark). The number of germinated seeds was recorded every day for 9 days. The germination rate (GR) was calculated as follows: GR (%) =h(Number of germinated seeds per dish/Number of total seeds per dish) × 100. Three replicate measurements were performed in each experiment, and the experiment was repeated three times.

For measurement of plant growth and yield, the germinated seeds (3 days after treatment) were planted in soil placed in plug tray (5 × 10 holes, 5 cm in diameter, 57 mm × 37 mm), and incubated in the growth chamber (25 °C, 50% moisture, 16 h light and 8 h dark). After 6 weeks, plants were harvested, and the height of individual plant was measured. Then, harvested plants were dried in an oven (60 °C) for 3 days, and the dry weight of individual plant was measured. For rice yield, the ripened grains were harvested after 16 weeks, and the number of grains harvested per individual plant was counted. Each experiment included 50 plants per treatment and was repeated three times.

### Analysis for disease resistance

Two-week-old rice seedlings grown from seeds in each treatment were used to examine resistance to a fungal disease caused by *Rhizoctonia solani*. Disease resistance was assessed by measuring disease severity and level of defense gene expression. To measure the disease severity, two-week-old rice seedlings were inoculated with the fungus by plugging barley seeds infected with *R*. *solani* AG-1 into soil as previously described^39^. Methods for inoculum preparation and inoculation of plants were well described in the previous study^39^. After growth for 4 weeks, the infection and disease severity of rice plants were assessed. The degree of disease symptoms was determined by visual inspections, two weeks after inoculation. Disease severity was determined on the following scale: 1 = less than 10% of leaf area with lesions, 2 = 10–25% of leaf area with lesions, 3 = 25–50% of leaf area with lesions, 4 = 50–75% of leaf area with lesions, and 5 = 75% or more severe lesion or dead leaves. The analysis was performed for total 60 plants per treatment from three replicate batches (20 plants per replicate).

The expression of pathogenesis related (PR) genes in rice plant (Table 3) was analyzed by RT-qPCR. Leaves were collected from two-week-old rice seedlings grown from seeds inoculated with plasma treated bacteria, non-treated bacteria, or water. Total RNA was extracted from the collected leaves using TRIZOL reagent (Invitrogen, Calsbad, CA, USA), and 5 µg RNA was used for the synthesis of complementary DNAs (cDNAs). cDNA was synthesized using the SMART® MMLV Reverse Transcriptase (Clontech, Mountain View, CA, USA), following the manufacturer’s instructions. A 1 µl of synthesized cDNA was used as template for qPCR. The qPCR was performed using iQ SYBR Green Supermix (Biorad, Hercules, CA, USA) and a Real-Time PCR detection system (CFX96, Biorad, Hercules, CA, USA). In all applications, the ubiquitin gene was used as a housekeeping gene (GenBank accession no. CA763279). The primer sequences for ubiquitin gene RT-PCR were as follows: forward 5′-CTCCCTGAGATTGCCCACAT-3′ and reverse 5′-CACGACTGGCAGCAACAAAT-3′. The thermal cycles for PCR followed a two-step protocol; activation at 95 °C for 3 min, followed by 40 cycles of denaturation at 95 °C for 10s and an annealing/extension at 60 °C for 30 s.

**Table 3:** List of PR gene specific primers used in the study.

Gene name	Accession no.	Gene function	Sequence (5′→3′)
OsPR3	D16221	endochitinase	F GCCACCGTCTCCTCAAGAC
			R GCGCTTGTAGAACCCAATGC
OsPR5	X68197	thaumatin-like protein	F TGATCGACGGCTACAACGTC
			R ATGGGCAGAAGACGACTTGG
LOX	D14000	lipoxygenase	F TCCACCGACGAGGAGTACCT
			R ATCAGCTGGTACGGCAGGAT

### Phytohormone analysis using Liquid Chromatography-Mass Spectrometry (LC-MS/MS)

Six-week-old rice plants infected by plasma treated bacteria, non-treated bacteria, or none were used for Indole Acetic Acid (IAA) and Salicylic Acid (SA) analysis. Five fresh rice plants were ground in liquid nitrogen using mortar and pestle. Ground powder was mixed with 5 ml of extraction solvent (methanol: isopropanol: glacial acetic acid = 20:79:1; v/v), and the mixture was stirred at 4 °C in the dark for 6 h. Then, the extraction mixture was centrifuged at 3,134 × *g* for 20 min, and supernatant was collected. The extraction steps were repeated twice, and the collected supernatant was dried in a vacuum evaporator (Jeiotech, Daejeon, Korea) for overnight. The dried residue was re-dissolved in 500 µl of methanol, and stored in vials at 4 °C, until LC-MS/MS analysis.

LC-MS/MS was performed for the quantitative analysis of IAA and SA using Ultimate 3000 RSLC HPLC system (Dionex, Germany) and Ion Trap Mass Spectrometry (Thermo Fisher Scientific, Waltham, MA, USA). In the HPLC analysis, TG-5 column (1.7 µm, 2.0 mm × 100 mm, Thermo Fisher Scientific, Waltham, MA, USA) was used and kept at 40 °C. For mobile phase, 0.1% Formic acid in water (A) and 0.1% Formic acid in acetonitrile (B) were used, with the flow rate of 300 µl per min. The gradient of mobile phase was as follows: 3 min of 95% A, 13 min of 100% B, 4 min of 95% A. MS/MS analysis for the quantification of IAA and SA was carried out through precursor scan using the selected ion monitoring (SIM) method in ion trap mass spectrometry. Electrospray ionization (ESI) was performed in positive or negative ion mode with the following settings: sheath gas (N_2_) 40 unit, aux gas (N_2_) 12 unit, spray voltage 3,500 V (positive ion mode) and −3,000 V (negative ion mode), H-ESI probe vaporizer temperature 200 °C, capillary temperature 350 °C, collision gas (argon) pressure 1.2 mTorr, collision energy 28 V. For quantification of the SA level, 137.3 negative mode molecular ion ([M-H]^−^) was used as precursor ion, and 93.2 product ion was monitored. For quantification of the IAA level, 130.1 positive mode ion was used as precursor ion, and 176.1 product ion was monitored.

### Scanning electron microscopy (SEM) analysis

Rice seeds inoculated by plasma treated bacteria, non-treated bacteria, or water were observed under scanning electron microscopy. Rice seeds were washed 3 times with 1 × PBS. Washed seeds were incubated in Karnovsky’s fixative (2% (v/v) paraformaldehyde and 2% (v/v) glutaraldehyde in 1 × PBS) at 4 °C for overnight. After washing seeds 3 times with PBS, secondary fixation was carried out by incubating seeds in 1% (v/v) osmium tetroxide for 2 h at room temperature in the dark. Seeds were washed twice with PBS, and then dehydrated by serial incubation in (30, 50, 70, 80, 90, and 100) % (twice) of ethanol, as previously described^55^. Dehydrated seeds were dried by twice incubating in hexamethyldisilazane (HMDS) for 15 min, and then mounted on the slide glass using nail polish. After coating with platinum once, seeds were examined under SEM (JSM 7001 F, JEOL, Tokyo, Japan).

### Visualization of bacteria in rice roots by confocal microscope

To examine the colonization pattern of bacteria *B*. *subtilis* CB-R05 within the rice seedling roots, the *gfp*-labeled *B*. *subtilis* CB-R05 strain constructed in the previous study^39^ was used. The *gfp*-labeled bacteria were treated with plasma for 3 min and inoculated onto roots of 2-week-old rice seedlings, as previously described^39^. After 24 h, confocal microscopic analysis was performed to confirm the presence/absence of *gfp*-tagged *B*. *subtilis* CB-R05 inside rice roots. Roots were cut into 1–2 cm lengths, placed onto slide glass, and then examined under confocal microscopy CLSM (Olympus Fluoview FV- 1000 MPE spectra, Tokyo, Japan), using an excitation wavelength of 488 nm. The light emitted was collected in the 500 to 600 nm range for *gfp* visualization. Root samples were observed by the depths of the x, y, and z axes, and thickness of 2.0 μm. Each experiment included three replicates, with 10 plants per treatment.

### Statistical analysis

All percentage and relative values of data set are represented as the mean ± standard deviation (S.D.) of the indicated number of replicates (≥3). Statistical analyses of the data were performed using student’s *t* test to establish the significance between data points, and significant differences were based on the *p* < 0.05 or *p* < 00.01 (*denotes p < 0.05, & **denotes p > 0.01).

## Supplementary information

Supplementary Information
