# Supplementary material for: Enhancement of vitality and activity of a plant growth-promoting bacteria (PGPB) by atmospheric pressure non-thermal plasma
Source: Sci Rep. 2019 Jan 31;9:1044. doi: 10.1038/s41598-018-38026-z (PMC6355859; doi:10.1038/s41598-018-38026-z)
Supplement: Supplementary file 1 — Supplementary Information [file 41598_2018_38026_MOESM1_ESM.pdf]

Supplementary Information

Enhancement of vitality and activity of a plant growth-promoting bacteria (PGPB) by atmospheric pressure non-thermal plasma

Sang-Hye Ji[1, 4], Ju-Sung Kim[1], Choong-Hwan Lee[2], Han-Sol Seo[2], Se-Chul Chun[3], Jaesung Oh[4], Eun-Ha Choi[1,5*], Gyungsoon Park[1,5*]

[1]Plasma Bioscience Research Center, Kwangwoon University, Seoul, 01897, Republic of Korea

[2]Department of Bioscience and Biotechnology, Konkuk University, Seoul 05029, Republic of Korea.

[3]Department of Bioresources and food science, College of Life and Environmental Sciences, Konkuk University, Seoul 05029, Republic of Korea

[4]Plasma Technology Research Center, National Fusion Research Institute, Gunsan-si, Jeollabuk-Do, 54004, Republic of Korea

[5]Department of Electrical and Biological Physics, Kwangwoon University, Seoul, 01897, Republic of Korea

* Corresponding author

Gyungsoon Park

Phone: +82-2-940-8324

Fax: +82-2-940-5664

Email: gyungp@kw.ac.kr

Eun-Ha Choi
Phone: +82-2-940-5236
Fax: +82-2-940-5664
Email: ehchoi@kw.ac.kr

Supplementary Methods

Primary metabolites analysis

Sample preparation for gas chromatography-time of flight-mass spectrometry (GC-TOF-MS) analysis Each bacteria pellet was subjected to extraction with 1 mL of 90% cold methanol (methanol:water, = 90:10, v/v) and 10 μL of internal standard (2-chlorophenylalanine, 0.5 mg/mL) on MM400 mixermill (Retsch®, Haan, Germany) for 5 min at RT with zirconium bead. The suspension was centrifuged at 15,000 rpm for 5 min. The supernatant was filtered through a 0.2 μm PTEE filters and then evaporated using a Speed Vacuum (Biotron, Seoul, Korea) for derivatization. Methoximation and silylation were performed for the GC-TOF-MS analysis. Methyloxime derivatives were obtained by dissolving the dry extracts in 50 μL of methoxyamine hydrochloride (20 mg/mL in pyridine) for 90 min at 30 °C. After methoximation, samples were silylated for 30 min at 37 °C by adding 50 μL of MSTFA, and then 1 μL of each derivatived sample was injected into GC-TOF-MS with splitless mode.

GC-TOF-MS analysis and data processing for multivariate statistical analysis GC-TOF-MS analysis was performed by using Agilent 7890 gas chromatograph system (Agilent Technologies, Palo Alto, CA, USA) with Agilent 7693 autosampler (Agilent Technologies) and Pegasus HT TOF MS (LECO, St. Joseph, MI, USA) system. Rtx-5MS column (i.d., 30 m X 0.25 mm, 0.25 μm particle size; Restek Corp., Bellefonte, PA, USA) was used with a constant flow of 1.5 mL/min of helium as carrier gas. The oven temperature was maintained at 75 °C for 2 min and then raised to 300 °C (15 °C increase per min), holding the temperature for 3 min. The temperatures of the front inlet and transfer line were 250 °C and 240 °C, respectively. The electron ionization was carried out at -70 eV and full scanning over a range of 50–1,000 m/z was used for mass data collection.

The GC-TOF-MS raw data files were converted into NetCDF format using the LECO Chroma TOF™ software (Version 4.44; LECO Corp.). After conversion, the peak detection, retention time, and alignment were processed by the Metalign software (http://www.metalign.nl), and the resulting data were exported to an Excel file. Multivariate statistical analysis was performed using the SIMCA-P+ (Version 12.0, Umetrics; Umea, Sweden). The significantly differed metabolites were selected by variable important in projection (VIP) > 0.7 by partial least squares-discriminant analysis (PLS-DA) dataset and *p*-value < 0.05 by analysis of variance (ANOVA). The box-whisker plots were conducted by the Statistica 7 software (StatSoft Inc., Tulsa, OK, USA).

Sequencing bacterial genome

PacBio RSII sequencing A sample of high-quality and high-molecular-weight DNA is required to prepare size- selected approximately 20 kb SMRTbell templates. We used NanoDrop spectrophotometer (Thermo Scientific, Waltham, MA, USA) and a Qubit fluorometer (Life Technologies, Waltham, MA, USA) to measure the concentration of genomic DNA. All samples passed screening QC criteria. For PacBio RS sequencing, 8 μg of input genomic DNA was used for 20 kb library preparation. For gDNA where the size range was less than 17 kb, we used the Bioanalyzer 2100 (Agilent) to determine the actual size distribution. The library insert sizes were in the optimal size range. gDNA was sheared with g-TUBE (Covaris Inc., Woburn, MA, USA) and purified using AMPure PB magnetic beads (Beckman Coulter Inc., Brea, CA, USA) if the apparent size was greater than 40 kb. The gDNA concentration was measured using both a NanoDrop spectrophotometer and a Qubit fluorometer, and approximately 200 ng/μL of gDNA was run on a field-inversion gel. Total 10μL library was prepared using PacBio DNA Template Prep Kit 1.0 (for 3–10 kb). SMRTbell

templates were annealed using PacBio DNA/Polymerase Binding Kit P6. The PacBio DNA Sequencing Kit 4.0 and 8 SMRT cells were used for sequencing. SMRT cells (Pacific Biosciences, Menlo Park, CA, USA) using C4 chemistry and 240 min movies were captured for each SMRT cell using the PacBio RS II (Pacific Biosciences) sequencing platform by Macrogen (Seoul, Korea). The subsequent steps are based on the PacBio Sample Net-Shared Protocol, which is available at http://pacificbiosciences.com.

Illumina Hiseq sequencing Each sequenced sample was prepared according to the Illumina protocols (TruSeq DNA Sample Prep Kit v2 Support (FC121-2001)). Briefly, 1 µg of genomic DNA was fragmented by covaris, the fragmented DNA was repaired, an ‘A’ was ligated to the 3′ end, Illumina adapters were then ligated to the fragments, and the sample was size selected aiming for a 400~500 base pair products. The size selected product was PCR amplified, and the final product was validated using the Agilent Bioanalyzer. Then, we sequenced using the HiSeq™ 4000 platform (Illumina, San Diego, USA)

Assembly, gene prediction/annotation Pacbio raw data were analyzed using SMRT Analysis Software (v 2.3). In this software, pacbio subreads were assembled by using HGAP3 protocol, to obtain assembled DNA sequence data. This assembled DNA sequence was corrected by Quiver (v1), the error correction step performed through SMRTpipe (v2.3.0.139497). In the next step after De Novo Assembly, HiSeq reads were applied for sequence compensation to construct contigs more accurately using Pilon (v1.21) tool. After correction step, Prokka was used for gene prediction and annotation. For additional annotation, the corrected sequences were searched against the GenBank non-redundant (NR) database using blastx (v2.4.0+).

Variant calling The filtered reads were mapped to reference genome using BWA. In this process, sufficient read depth was needed for more accurate analysis. After mapping, duplicated reads were removed, and variants (SNPs and short indels) were captured through aligned reads' information using by SAMTools. The variants were classified by each chromosomes or scaffolds, and the information of the location was marked.

Supplementary Table S1. Summary of 2-D gel electrophoresis

Total number of protein spots	[1]B gel: 518 [2]PB gel: 585
Number of spots paired in B and PB gel	436
Number of spots non-paired in B and PB gel	231
Number of spots with increased intensity more than 2-fold in PB gel compared to B gel	38
Number of spots with decreased intensity more than 2-fold in PB gel compared to B gel	57

[1] 2-dimentional electrophoretic gel for proteins extracted from bacteria (non-plasma treated bacteria)

[2] 2-dimentional electrophoretic gel for proteins extracted from plasma treated bacteria

Supplementary Table S2. Spots with different intensity in PB (Plasma treated bacteria) compared to B (Non-plasma treated bacteria) gel

More than 2-fold increase in intensity in PB				More than 2-fold decrease in intensity in PB			
Spot ID number	B (% vol)	PB (% vol)	Ratio (PB/B)	Spot ID number	B (% vol)	PB (% vol)	Ratio (PB/B)
69	0.457	0.909	2	53	0.056	0.027	2.1
82	0.112	0.315	2.8	122	0.192	0.097	2
88	0.02	0.08	3.9	134	0.234	0.117	2
110	0.058	0.123	2.1	142	0.13	0.047	2.7
114	0.062	0.15	2.4	150	0.388	0.076	5.1
121	0.062	0.199	3.2	151	0.134	0.066	2
133	0.025	0.05	2	153	0.17	0.054	3.1
152	0.058	0.24	4.1	192	0.118	0.032	3.7
167	0.029	0.058	2	202	0.183	0.084	2.2
171	0.032	0.095	3	233	0.094	0.043	2.2
179	0.044	0.104	2.4	236	0.312	0.111	2.8
186	0.079	0.229	2.9	254	0.32	0.139	2.3
214*	0.103	0.366	3.5	257	0.447	0.228	2
225	0.163	0.355	2.2	298	0.066	0.028	2.3
232	0.065	0.337	5.2	300	0.163	0.04	4.1
241	0.135	0.275	2	314	0.302	0.147	2.1
250	0.036	0.073	2.1	325	0.852	0.189	4.5
260	0.034	0.599	17.5	328	0.863	0.422	2
279	0.032	0.082	2.6	332	0.189	0.082	2.3
291	0.042	0.108	2.5	341	0.104	0.051	2
339	0.084	0.375	4.5	343	0.211	0.068	3.1
355	0.043	0.086	2	354	0.607	0.301	2
357	0.025	0.056	2.2	356	0.109	0.055	2
373	0.097	0.321	3.3	390	0.147	0.035	4.2
376	0.056	0.117	2.1	440	0.078	0.028	2.8
426	0.094	0.187	2	455	0.392	0.188	2.1
447	0.021	0.236	11.3	459	0.082	0.041	2
516	0.079	0.173	2.2	476	1.485	0.4	3.7
557	0.039	0.134	3.4	480	0.162	0.039	4.1
642	0.076	0.236	3.1	482	0.176	0.061	2.9
660	0.067	0.147	2.2	484	0.854	0.186	4.6
764	0.479	1.078	2.3	493	0.095	0.048	2
765	0.222	0.95	4.3	495	0.392	0.11	3.6
796	0.437	1.242	2.8	509	0.104	0.05	2.1
987	0.045	0.133	2.9	512	0.131	0.035	3.8
988	0.029	0.075	2.6	558	0.239	0.08	3
989	0.024	0.048	2	562	0.411	0.169	2.4
1011	0.033	0.065	2	568	0.06	0.03	2
				596	0.22	0.096	2.3
				611	0.265	0.129	2
				620	0.349	0.09	3.9
				621	0.097	0.038	2.5
				628	0.209	0.055	3.8
				662	0.413	0.205	2

	664	0.107	0.043	2.5
	687	0.509	0.129	3.9
	697	0.08	0.041	2
	710	0.222	0.049	4.5
	739	0.223	0.042	5.3
	754	0.313	0.109	2.9
	827	0.149	0.07	2.1
	838	0.191	0.093	2
	855	0.102	0.045	2.3
	1001	0.033	0.017	2
	1007	0.064	0.025	2.6
	1008	0.041	0.014	3
	1010	0.03	0.013	2.3

* Underlined numbers represent protein spots of which level is increased or decreased more than 3-fold. These spots were further analyzed for protein identification using nano LS-MS/MS.

Supplementary Table S3. Spots found only in B (non-plasma treated bacteria) gel

Spot ID number	Area	Vol	%Vol	Spot ID number	Area	Vol	%Vol
50	6.82154	538.08	0.0267688	410	9.33253	1111.31	0.0552863
54	5.94269	226.521	0.0112692	414	7.00289	817.409	0.0406651
56	7.22608	639.959	0.0318372	420	9.29068	850.535	0.0423131
72	5.88689	658.327	0.032751	430	10.9647	2338.43	0.116334
89	5.23124	380.287	0.0189188	443	10.7554	1195.1	0.0594548
92	16.7121	6127.42	0.304832	453	12.5829	2617.78	0.130231
94	10.03	2174.58	0.108183	461	8.64898	642.362	0.0319567
98	6.57044	900.783	0.0448129	462	0.01395	0	0
101	10.1835	1607.89	0.0799905	468	8.09098	1700.74	0.0846096
102	16.3912	3331.3	0.165728	471	12.276	3109.93	0.154715
111	16.7818	3271.06	0.162731	490	23.7289	2852.38	0.141902
115	9.15118	633.371	0.0315094	506	9.51388	1367.25	0.0680192
123	10.4206	1102.41	0.0548434	518	15.8751	1877	0.0933783
138	7.11449	689.681	0.0343108	525	10.6996	633.636	0.0315226
140	10.2811	2160.96	0.107505	526	12.9037	1505.29	0.0748861
161	9.17908	3696.04	0.183874	530	9.96028	1372.23	0.0682668
176	8.59318	702.638	0.0349554	535	16.4749	2101.76	0.10456
180	8.38393	1782.38	0.0886712	559	14.5777	1534.43	0.0763359
197	8.74663	1977.23	0.0983646	573	25.0123	9749.38	0.48502
201	12.4434	2311	0.114969	576	8.76058	1803.31	0.0897127
203	9.37438	1044.11	0.0519433	583	10.5183	2729.16	0.135773
205	7.71433	605.06	0.030101	586	13.7407	2060.98	0.102531
215	5.42654	614.538	0.0305725	602	7.95148	695.018	0.0345763
222	12.6805	1857.66	0.0924164	634	14.4382	1343.91	0.0668577
227	6.76574	1884.12	0.0937326	650	19.6695	3774.4	0.187772
228	17.298	5806.41	0.288862	652	13.5873	1282.27	0.0637913
230	9.96028	2266.12	0.112737	668	14.6754	4008	0.199393
234	9.13723	1221.4	0.0607633	683	23.6731	4687.97	0.233221
244	6.34724	1574.85	0.078347	694	18.5674	4931.11	0.245317
258	21.1063	7537.21	0.374967	696	14.0616	1604.46	0.0798197
264	8.11888	2105.76	0.104759	711	24.1613	4599.61	0.228825
276	16.8516	5303.43	0.263839	733	14.5917	493.961	0.024574
286	12.8479	5897.03	0.29337	734	19.8229	2295.02	0.114175
288	7.56088	699.361	0.0347924	749	20.7715	3269.78	0.162667
296	10.2811	2002.86	0.0996397	775	16.6981	3613.63	0.179774
297	12.7224	3354.84	0.166899	813	15.903	2743.47	0.136484
335	17.1864	8793.2	0.437451	823	18.3303	3407.61	0.169525
351	9.58363	3050.57	0.151762	852	29.839	13071.9	0.650313
366	11.6343	2014.03	0.100196	996	10.4485	1314.2	0.0653798
367	12.1783	3880.36	0.193043	997	13.3501	2353.96	0.117107
402	9.02563	3224.48	0.160414	1027	7.93753	642.87	0.031982

Supplementary Table S4. Spots found in only PB (Plasma treated bacteria) gel

Spot number	Area	Vol	%Vol	Spot number	Area	Vol	%Vol
65	15.7914	788.894	0.0289341	555	16.0146	3472.47	0.127359
68	8.38393	944.455	0.0346396	560	14.4103	523.109	0.019186
77	10.1556	1168.04	0.0428402	571	12.8898	2148.76	0.0788096
94	8.06308	766.104	0.0280983	577	13.2804	2491.86	0.0913937
103	6.36119	572.646	0.0210029	578	19.2649	2525.02	0.0926096
104	11.9412	1461.13	0.0535898	580	11.0484	1329.07	0.0487461
116	9.12328	1266.79	0.046462	581	16.2238	3703.3	0.135825
118	8.35603	1316.67	0.0482912	583	9.91843	823.501	0.0302034
127	10.3369	934.698	0.0342818	587	11.1739	1794.9	0.0658313
141	13.8384	3139.31	0.11514	591	8.25838	557.511	0.0204477
142	9.69523	3003.57	0.110161	592	11.8017	2786.55	0.102202
145	10.6717	1737.97	0.0637433	593	28.4161	7372.56	0.270402
153	11.7877	1235.49	0.045314	594	16.0983	1693.83	0.0621245
167	9.06748	1903.31	0.0698072	598	13.9779	1508.61	0.0553309
170	14.8707	2937.4	0.107735	605	20.0043	5014.32	0.18391
179	8.81638	362.474	0.0132944	606	10.8391	2784.01	0.102109
186	9.79288	1027.27	0.0376769	612	18.0094	4668.98	0.171243
205	11.1321	997.395	0.0365813	622	13.2246	2579.61	0.0946118
250	24.8309	8024.43	0.294311	641	15.052	2927.96	0.107388
251	12.3597	3385.7	0.124177	665	25.933	5026.75	0.184365
252	17.6886	4094.06	0.150157	667	17.6049	1645.81	0.0603633
254	33.4241	7493.16	0.274825	671	30.104	5853.76	0.214697
256	16.7121	5674.5	0.208123	676	13.9639	1308.41	0.0479883
261	10.323	1393.37	0.0511042	692	15.7216	3292.85	0.120771
266	16.4052	3043.71	0.111634	695	14.2429	2477.99	0.0908848
270	14.8009	4521.68	0.165841	697	20.5483	2736.15	0.100353
274	11.7738	1289.26	0.0472859	700	23.5476	3924.18	0.143927
284	14.9125	4616.6	0.169322	704	15.9588	2250.38	0.082537
293	12.7642	1739.02	0.0637817	711	19.4602	2185.48	0.0801567
295	9.03958	4316.97	0.158333	716	12.6666	2170.25	0.079598
296	8.42578	555.81	0.0203853	718	11.2995	1043.29	0.0382646
301	5.70554	1235.2	0.0453033	721	22.4316	4063.25	0.149027
306	13.3641	1624.03	0.0595642	722	21.0366	3514.93	0.128916
308	14.5359	2719.95	0.0997593	725	21.4969	4808.51	0.176361
314	33.7729	20046.6	0.735245	726	19.1254	3111.95	0.114136
325	14.3266	1618.63	0.0593662	739	13.3083	2007.39	0.0736246
335	8.98378	1002.06	0.0367526	742	19.9903	5140.02	0.18852
341	14.5219	1632.82	0.0598867	743	36.9256	9168.73	0.33628
342	9.19303	2707.74	0.0993113	746	15.4566	2796.29	0.102559
343	16.7679	5009.63	0.183737	756	22.8919	3145.22	0.115357
346	10.7554	1536.06	0.056338	762	16.6842	3577.64	0.131216
355	6.64019	1000.98	0.0367127	763	25.5284	4212.95	0.154518
358	6.24959	862.414	0.0316306	767	20.3112	3408.91	0.125028
360	8.90008	951.514	0.0348985	777	18.414	2721.54	0.0998175
362	13.6291	2623.64	0.0962269	781	27.593	4792.31	0.175767
365	10.6717	534.507	0.019604	782	35.4887	9287.86	0.34065
377	12.6108	2346.62	0.0860667	787	19.3486	3378.1	0.123898
389	11.7738	2381.33	0.0873397	788	23.2546	5836.24	0.214055

391	14.2987	3950.13	0.144878	813	21.9852	2247.02	0.0824136
401	12.834	1506.64	0.0552588	817	20.2554	2795.87	0.102544
406	11.5506	2315.15	0.0849124	825	15.9867	985.717	0.036153
408	7.93753	1169.44	0.0428913	836	15.7216	1439.48	0.0527957
409	12.1923	1595.18	0.0585063	846	34.3867	7057.67	0.258853
415	12.6666	4298.23	0.157645	848	32.4476	9135.96	0.335078
421	10.6996	1853.17	0.0679685	855	16.5447	3021.35	0.110814
422	19.0557	2730.01	0.100128	869	18.1908	1069.14	0.0392128
424	16.2517	2971.92	0.109001	887	15.9867	1798.22	0.0659531
431	6.62624	1250.77	0.0458745	891	25.7237	15224.9	0.5584
432	10.588	2610.9	0.0957596	918	15.0241	966.772	0.0354582
438	11.8854	2173.71	0.0797249	935	13.4199	1319.03	0.0483777
450	14.0755	6273.75	0.230101	938	18.6651	2819.85	0.103423
454	13.6989	2422.8	0.0888606	940	10.6857	825.865	0.0302901
455	13.7268	1369.53	0.0502301	942	8.38393	968.853	0.0355345
458	16.6842	5517.02	0.202347	943	14.787	1978.01	0.072547
465	13.2385	2604.73	0.0955333	967	12.4852	759.268	0.0278476
474	21.8875	5838.1	0.214123	1025	13.3501	863.419	0.0316675
480	13.2804	975.214	0.0357678	1027	9.16513	697.122	0.0255682
485	25.2494	14005.6	0.513681	1028	9.16513	618.43	0.0226821
489	10.4206	1855.3	0.0680467	1032	10.4485	1146.48	0.0420492
492	10.4206	1195.38	0.0438426	1036	13.3501	1600.84	0.0587139
514	17.577	7860.69	0.288305	1039	9.91843	1532.61	0.0562114
518	13.8663	1179.41	0.0432572	1042	7.93753	750.927	0.0275416
531	12.8898	1635.3	0.0599776	1048	10.4485	988.355	0.0362498
533	12.8479	1493.29	0.0547691	1050	18.3721	1865.84	0.0684332
541	14.0895	1099.58	0.0403291				

Supplementary Table S5. Primary metabolites* identified by GC-TOF-MS analysis

Peak	Tentative identification	RT (min)	Mass fragment pattern	Unique Mass	TMS	ID
Organic acids						
**1**	**Succinic acid**[ab]**	**7.68**	**148, 174, 149, 247, 129, 107, 133**	**148**	**2**	**STD/MS**
2	Lactic acid	5.2	117, 146, 148, 191, 234, 190, 133	117	2	STD/MS
Alcohols						
3	Propanediol	5.13	115, 130, 151, 148, 177, 149, 133	115	2	MS
4	Glycerol alpha-monochlorohydrin[a]	6.51	116, 101, 151, 103, 148, 117, 131	116	2	MS
**5**	**Glycerol**[ab]	**7.34**	**117, 205, 103, 133, 148, 218, 149**	**117**	**3**	**STD/MS**
Lipids						
6	Hydroxybutyric acid[a]	6.94	148, 174, 117, 189, 149, 233, 133	148	2	MS
7	Pentadecanoic acid	12.4	117, 129, 132, 145, 131, 299, 118	117	1	MS
8	Palmitic acid[a]	13.22	117, 132, 129, 145, 131, 313, 118	117	1	STD/MS
**9**	**Oleanitrile**[ab]	**13.51**	**122, 136, 150, 110, 123, 108, 137**	**122**	**0**	**MS**
**10**	**Stearic acid**[ab]	**14.41**	**117, 132, 129, 131, 145, 116, 133**	**117**	**1**	**STD/MS**
**11**	**Oleamide**[ab]	**15.42**	**131, 144, 116, 128, 198, 145, 115**	**131**	**1**	**STD/MS**
Etc.						
12	Borate	4.44	221, 222, 263, 133, 223, 175, 191	221	3	MS
13	Phenol[a]	5.14	151, 207, 166, 115, 130, 152, 148	151	1	MS
14	Hydroxylamine	5.78	133, 146, 119, 249, 130, 100, 134	133	3	STD/MS
**15**	**Cyclohexenone**[ab]	**5.91**	**138, 139, 123, 110, 115, 108, 124**	**138**	**0**	**MS**
16	Urea	6.96	189, 171, 148, 149, 100, 190, 174	189	2	STD/MS
17	Benzoinc acid	7.09	179, 105, 135, 80, 136, 194, 106	179	1	MS
**18**	**Phosphoric acid**[ab]	**7.37**	**299, 133, 300, 314, 301, 193, 207**	**299**	**3**	**STD/MS**
19	1,1,1-Tris(hydroxymethyl)propane	8.47	174, 191, 113, 110, 100, 175, 156	174	3	MS
Non-identifications						
20	N.I.1	5.63	105, 136, 204, 103, 148, 116, 106	105	-	-
21	N.I.2	5.65	204, 148, 149, 131, 205, 133, 105	204	-	-
22	N.I.3[a]	6.03	133, 148, 100, 220, 146, 235, 160	133	-	-
23	N.I.4	7.64	107, 256, 140, 120, 118, 186, 153	107	-	-
24	N.I.5	7.95	184, 134, 285, 100, 185, 135, 118	184	-	-
25	N.I.6[a]	9.43	180, 110, 200, 101, 127, 270, 103	180	-	-
26	N.I.7[a]	9.74	263, 264, 278, 265, 115, 207, 175	263	-	-
27	N.I.8	11.06	154, 146, 242, 130, 156, 157, 118	154	-	-
28	N.I.9[a]	14.26	160, 314, 130, 117, 315, 144, 161	160	-	-
**29**	**N.I.10**[ab]	**14.31**	**131, 144, 116, 128, 117, 115, 145**	**131**	**-**	**-**
30	N.I.11[a]	15.69	314, 160, 358, 117, 204, 130, 100	314	-	-
31	N.I.12	17.45	131, 144, 116, 128, 132, 115, 145	131	-	-

*, Total 31 primary metabolites were identified;
Organic acids: 2, Alcohols: 3, Lipids: 6, Etc: 8, Non-identifications: 12.
**, Bold font indicates metabolites that show significant differences between B (non-plasma

treated bacteria) and PB (plasma treated bacteria) groups

a, VIP1, 2 > 0.7

b, *P*-value < 0.05)

Supplementary Table S6. Assessment of disease severity and percentage of dead leaves

Disease severity (1-5)							
**Plant number**	**Control**	**B**	**PB**	**Plant number**	**Control**	**B**	**PB**
1	5	5	5	31	3	1	5
2	5	5	4	32	3	3	5
3	5	5	3	33	4	3	3
4	4	5	2	34	2	3	3
5	4	5	2	35	1	2	4
6	4	4	3	36	2	3	2
7	3	3	2	37	2	2	3
8	2	3	1	38	2	3	3
9	5	2	3	39	5	3	4
10	3	3	2	40	5	2	2
11	4	5	5	41	4	3	4
12	2	5	4	42	4	3	3
13	3	4	2	43	5	2	3
14	3	4	3	44	4	1	3
15	4	4	4	45	5	2	4
16	5	4	4	46	5	3	2
17	5	4	4	47	4	2	1
18	5	4	3	48	3	3	3
19	5	3	5	49	4	2	2
20	4	3	3	50	4	5	1
21	3	5	2	51	4	2	1
22	3	5	5	52	2	2	2
23	2	5	1	53	2	1	2
24	3	4	1	54	3	3	1
25	4	3	3	55	5	2	1
26	4	3	2	56	3	3	2
27	5	3	5	57	4	3	4
28	5	3	5	58	4	1	2
29	3	2	4	59	4	1	1
30	4	2	4	60	5	3	1
**Average**	3.72	3.12	2.88				
**Standard deviation.**	1.09	1.19	1.29				

Total number of dead leaves		
**Control**	**B**	**PB**
17	11	8
**Average percentage of dead leaves in individual plant (%); (total number of dead leaves / total number of plants (60)) x 100**		
**Control**	**B**	**PB**
28.3	18.3	13.3

Control: water inoculated rice seedlings, **B**: rice seedlings infected with untreated bacteria, **PB**: rice seedlings infected with plasma treated (air plasma for 3 min) bacteria.

Supplementary Table S7. Results of sequencing for bacterial genome

Chromo-some	Position	Ref[1]	Alt[2]	Quality[3]	Hom/ Het[4]	Read Depth[5]	Alt Depth[6]	Gene Name	Gene ID	Start	End
contig1	614681	G	T	134	het	243	53	spo0B	00704	614509	615087
contig1	1621694	T	C	222	het	280	99	spo0F	01737	1621485	1621859
contig1	4224357	G	C	225	hom	185	185	cwlS	04342	4223490	4224734

1 Reference sequence regarding specific position
2 DNA sequence of the sample
3 Phred-scaled probability of all samples being homozygous reference. (The value is in-log. The smaller the value, the more likely ALT is wrong.)
4 Shows genotype "hom" means non-reference homozygote, "het" means heterozygote.
Homozygous: The circumstances when there are mutations on most reads that are mapped to certain region.
Heterozygous: The circumstances when there are mutations on some reads that are mapped to certain region.
5 Total read depth.
6 Allelic depths for the ref and alt alleles in the order listed.

a

B gel PB gel

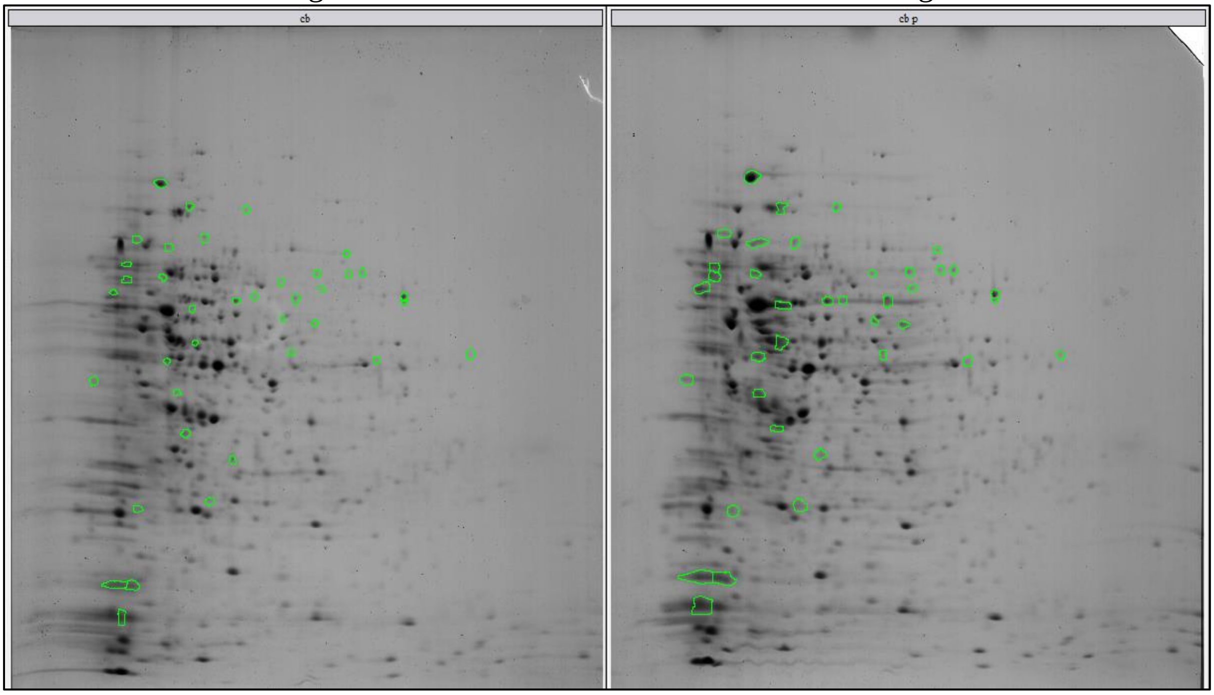

b

B gel PB gel

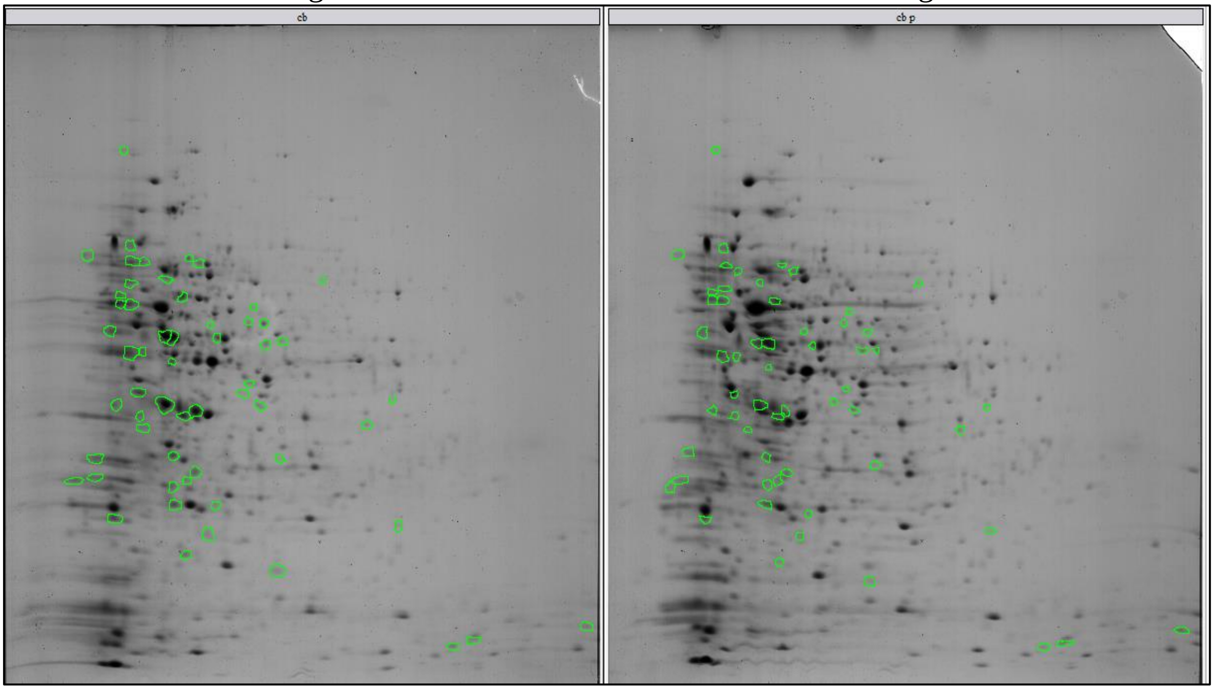

Supplementary Figure S1. **a.** Group spots showing more than 2-fold increased intensity in PB (Plasma treated bacteria) compared to B (bacteria) gel. **b.** Group spots showing more than 2-fold decreased intensity in PB compared to B gel

B gel PB gel

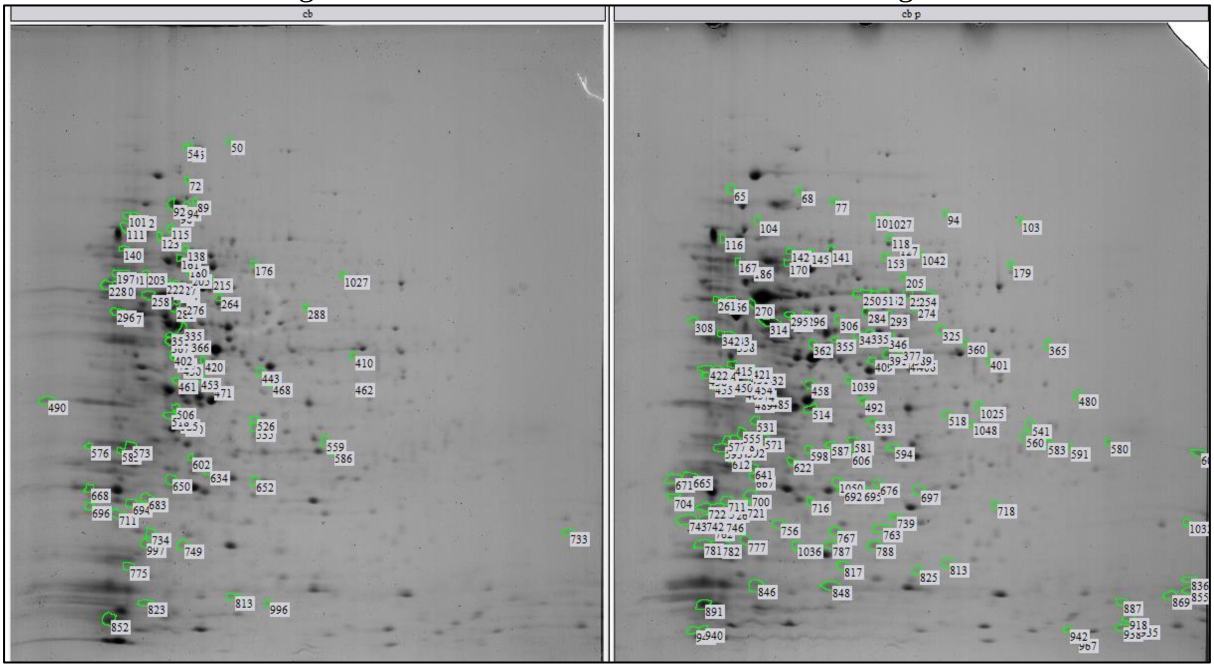

Supplementary Figure S2. Group spots shown in only PB (Plasma treated bacteria) gel and in only B (bacteria) gel.

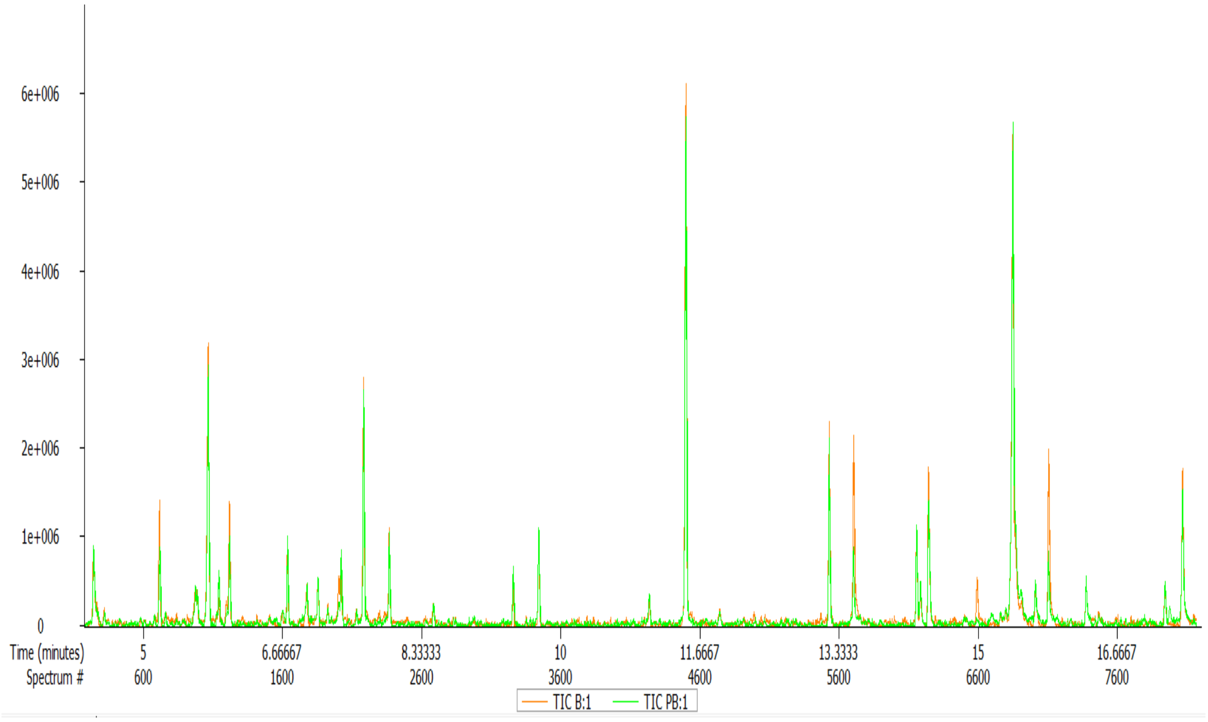

Supplementary Figure S3. GC-TOF-MS chromatogram of primary metabolites extracted from B (bacteria) and PB (plasma treated bacteria). Orange and green lines represent bacteria and plasma treated bacteria sample, respectively.

a

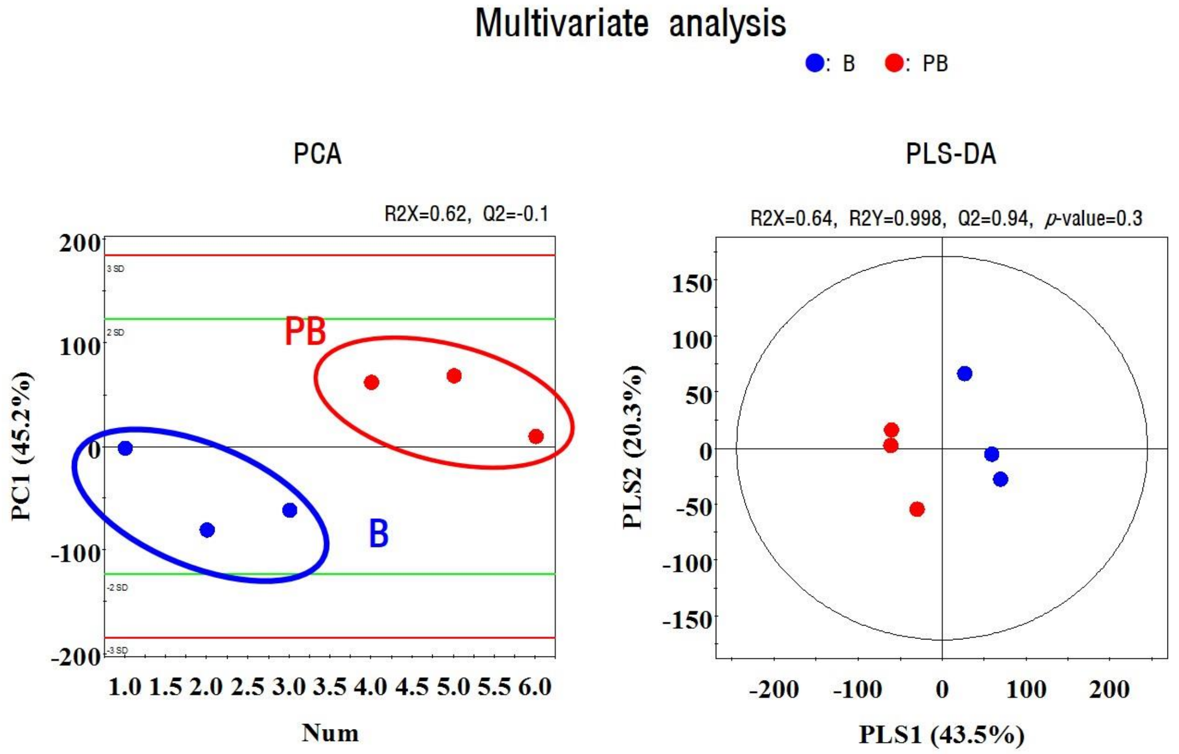

b

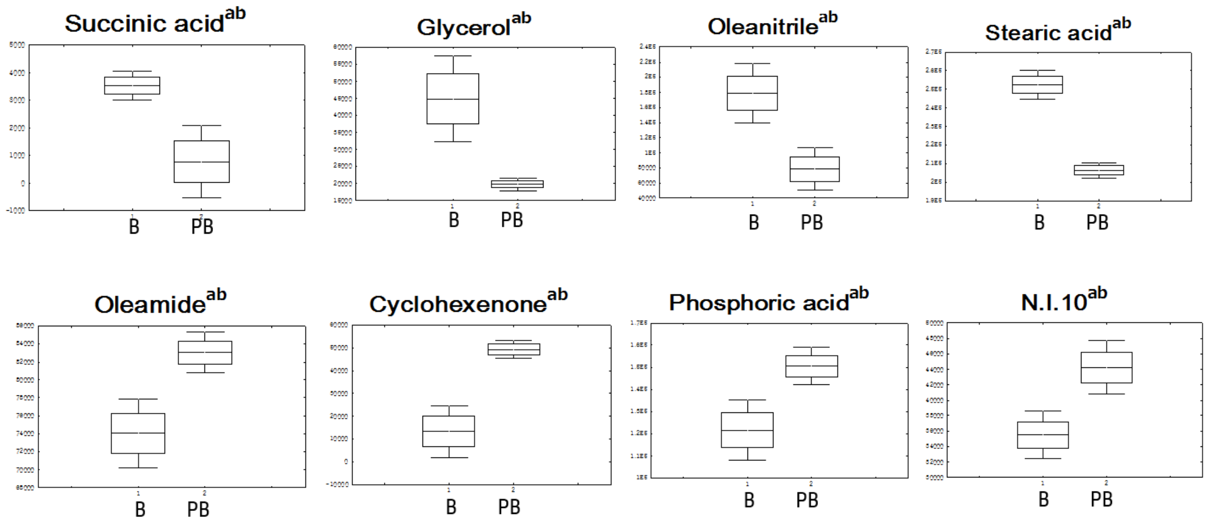

Supplementary Figure S4. **a.** Plots of PCA and PLS-DA analysis. **b.** Primary metabolites that show significant differences in amount between B (bacteria) and PB (plasma treated bacteria) groups. a, VIP1, 2 > 0.7 b, P-value < 0.05)
